# Integrating Anti-SARS-CoV-2 Spike Protein IgG, IL-6, and NK Cell Percentage with Routine Laboratory Markers for COVID-19 Severity Assessment: Development and Validation of the CORIS Nomogram

**DOI:** 10.3390/diagnostics16193133

**Published:** 2026-09-26

**Authors:** Yihe Zhang, Xuanming Xu, Lian Tang, Jiayue Ma, Yajie Wang

**Affiliations:** 1 National Key Laboratory of Intelligent Tracking and Forecasting for Infectious Diseases, Beijing Key Laboratory of Viral Infectious Diseases, Department of Clinical Laboratory, Beijing Ditan Hospital, Capital Medical University, Beijing 100015, China; 2 School of Medical Technology, Capital Medical University, Beijing 101300, China; 3 Department of Neurology, Beijing Tiantan Hospital, Capital Medical University, Beijing 100070, China

**Keywords:** SARS-CoV-2, COVID-19, immune biomarkers, anti-SARS-CoV-2 spike protein IgG, interleukin-6, nomogram

## Abstract

**Background:** Rapid assessment of coronavirus disease 2019 (COVID-19) severity at hospital admission is essential for clinical triage and efficient healthcare resource allocation. However, prediction models developed using data from the early phases of the pandemic may exhibit reduced performance owing to the continued evolution of severe acute respiratory syndrome coronavirus 2 (SARS-CoV-2) variants, and existing nomograms rarely integrate routine laboratory parameters with immune-related biomarkers in a systematic manner. **Methods:** We retrospectively included 774 patients with laboratory-confirmed COVID-19 admitted to Beijing Ditan Hospital between January 2022 and December 2023, and randomly assigned them at a 7:3 ratio to a training set (*n* = 543) and a validation set (*n* = 231). Variables consistently selected by three approaches—multivariable logistic regression, least absolute shrinkage and selection operator (LASSO) regression, and recursive feature elimination (RFE)—were defined as core predictors and used to construct the COVID Routine-Immune Score (CORIS) nomogram. Model performance was assessed in terms of discrimination, calibration, and clinical net benefit. The prespecified outcome was non-severe versus severe/critical status determined at hospital admission. CORIS was designed for admission severity discrimination and was not intended to diagnose SARS-CoV-2 infection or predict post-admission deterioration, survival, or thrombotic events. **Results:** Age, lactate dehydrogenase (LDH), anti-SARS-CoV-2 spike protein immunoglobulin G (IgG), interleukin-6 (IL-6), and NK cell percentage (NK-pct) were consistently identified by all three feature-selection methods. The CORIS nomogram achieved an area under the receiver operating characteristic curve (AUC) of 0.914 (95% CI, 0.884–0.940) in the training set and 0.918 (95% CI, 0.868–0.960) in the validation set, with good calibration (Hosmer–Lemeshow *p* = 0.322 and 0.659, respectively). In the validation set, the CORIS nomogram significantly outperformed a lymphocyte subset-based model (AUC, 0.811; DeLong *p* < 0.001) and a routine laboratory parameter-based model (AUC, 0.839; DeLong *p* = 0.003). **Conclusions:** The CORIS nomogram integrates predictors spanning four dimensions—demographic susceptibility, tissue injury, systemic inflammation, and immune status—to differentiate non-severe from severe or critical COVID-19. The model demonstrated favorable discrimination and calibration, and may provide an objective and practical tool for severity assessment at hospital admission.

## 1. Introduction

Coronavirus disease 2019 (COVID-19), caused by severe acute respiratory syndrome coronavirus 2 (SARS-CoV-2), has resulted in hundreds of millions of confirmed cases and millions of deaths worldwide since it was first reported in December 2019 [1,2]. Although COVID-19 management has become increasingly routine, SARS-CoV-2 continues to circulate globally, and severe disease and its associated burden on healthcare resources remain major challenges for health systems worldwide [3]. Clinically, COVID-19 presents with a broad spectrum of manifestations, ranging from asymptomatic infection and mild illness to severe or critical disease and death [4]. Patients with severe or critical COVID-19 are at increased risk of life-threatening complications, including acute respiratory distress syndrome and multiple organ dysfunction, and often require prompt intensive care and specialized treatment [4,5]. Therefore, accurate assessment of disease severity and early differentiation of non-severe from severe or critical cases at hospital admission are essential for optimizing clinical triage, facilitating efficient allocation of healthcare resources, and guiding individualized treatment strategies [6].

Objective and quantitative assessment tools are essential for the early evaluation of COVID-19 severity. However, SARS-CoV-2 has continued to evolve throughout the pandemic, giving rise to successive variants, including Alpha, Delta, Omicron, and numerous Omicron sublineages. These variants differ substantially in viral fitness, pathogenicity, tissue tropism, immune evasion, and associated clinical manifestations [7,8]. Consequently, prediction models developed using data from earlier variant-dominant periods may exhibit reduced discrimination and poor calibration when applied to populations infected with newly emerging variants. Systematic reviews and large-scale external validation studies have further shown that the performance of COVID-19 prediction models varies considerably across patient populations, healthcare settings, and phases of the pandemic [6,9]. Hence, severity assessment tools must be developed and regularly updated using contemporary clinical data encompassing multiple SARS-CoV-2 variant-dominant periods.

A nomogram is an intuitive graphical decision-support tool that translates a complex regression model into a practical and readily interpretable scoring system. Nomograms have been widely applied across a broad range of diseases to support individualized risk prediction, risk stratification, and prognostic assessment. In COVID-19, nomograms can integrate multidimensional clinical variables to assess severe or critical status at hospital admission. These tools may therefore facilitate clinical triage, inform treatment decisions, and support the efficient allocation of healthcare resources [10,11].

In selecting laboratory predictors, it is important to consider the central roles of inflammation and immune dysregulation in COVID-19 progression. Elevated levels of inflammatory and tissue injury biomarkers, including C-reactive protein (CRP), interleukin-6 (IL-6), and lactate dehydrogenase (LDH), have consistently been associated with greater disease severity and poorer clinical outcomes [12,13]. From an immunological perspective, lymphopenia is a hallmark of severe COVID-19, while the numbers and functional states of T-cell subsets, natural killer (NK) cells, and B cells may also be altered during SARS-CoV-2 infection [14,15]. However, most published COVID-19 severity nomograms have the following limitations. The model developed by Li et al. (2021) combines sex and age with absolute T-cell, B-cell, and helper T-cell counts; because it was developed during the early phase of the pandemic, the transportability of this predictor set to populations affected by later variants remains uncertain [11]. The model developed by Chen et al. (2024), which is based on routine laboratory parameters, encompasses the dimensions of inflammation and tissue injury but does not incorporate immune-related biomarkers, potentially omitting the critical information conveyed by immune dysregulation [10,11]. Overall, existing nomograms are either confined to a single category of indicators or have yet to achieve multidimensional integration of inflammation, immunity, and tissue injury.

The present study aimed to address these limitations in three ways. First, for variable selection, three complementary approaches—multivariable logistic regression, LASSO regression, and recursive feature elimination (RFE)—were applied and only variables selected by all three methods were retained to reduce the selection bias inherent in any single method. Second, immune-related markers, including anti-SARS-CoV-2 spike protein immunoglobulin G (IgG) and NK cell percentage (NK%), were introduced and combined with routine clinical parameters, such as age, lactate dehydrogenase (LDH), and IL-6, to construct a multidimensional model—the COVID Routine-Immune Score (CORIS) nomogram—for individualized discrimination between non-severe and severe or critical COVID-19, thereby providing an objective and practical basis for clinical decision-making. Third, to enhance the contemporary relevance of the data, the model was constructed using clinical data from an Omicron-dominant epidemic period.

## 2. Materials and Methods

### 2.1. Study Design and Population

We conducted a retrospective analysis of consecutive adults (≥18 years) hospitalized with laboratory-confirmed SARS-CoV-2 infection at Beijing Ditan Hospital between January 2022 and December 2023. The study period fell entirely within the Omicron-dominant epidemic period: early 2022 was predominantly characterized by BA.1/BA.2 sublineages; beginning in July 2022, BA.5.2 became the dominant strain; from late 2022 to early 2023, BA.5.2 and BF.7 sublineages predominated; thereafter, multiple Omicron sublineages continued to co-circulate. Infection was verified by reverse-transcription PCR or antigen testing. Disease severity was stratified according to the Chinese national Diagnosis and Treatment Protocols for COVID-19 (Trial Versions 9 and 10): patients with non-severe illness were defined as those with mild clinical manifestations who did not meet the criteria for severe or critical disease. Patients with severe or critical illness were required to meet at least one of the following criteria that could not be attributed to causes other than SARS-CoV-2 infection: (1) dyspnea with a respiratory rate ≥30 breaths/min; (2) finger oxygen saturation ≤93% on room air at rest; (3) arterial partial pressure of oxygen to fraction of inspired oxygen ratio (PaO_2_/FiO_2_) ≤ 300 mmHg; (4) progressive worsening of clinical symptoms with >50% progression on pulmonary imaging within 24–48 h; (5) respiratory failure requiring mechanical ventilation; (6) shock; or (7) requirement for intensive care unit (ICU) monitoring and treatment. Full eligibility criteria and the patient selection process are depicted in Figure 1. A total of 774 patients were enrolled, comprising 646 non-severe (83.46%) and 128 severe/critical cases (16.54%). The institutional review board of Beijing Ditan Hospital approved the study (DTEC-KY2022-052-02; DTEC-KY2021-010-01) and waived the requirement for informed consent because of the retrospective study design. All procedures were conducted in accordance with the Declaration of Helsinki. The outcome was the non-severe versus severe/critical status documented at the admission assessment; patients were not reclassified according to subsequent in-hospital deterioration, survival, or thrombotic events. The imaging-progression criterion was applied only when progression had already been documented as part of the admission assessment and was not based on changes occurring after admission.

### 2.2. Data Collection

The following data, obtained within 24 h of hospital admission, were extracted from the electronic medical record system: (1) demographic characteristics, including age and sex; (2) medical history, including smoking, alcohol consumption, diabetes mellitus, hypertension, and hyperlipidemia; (3) anthropometric measurements, including body mass index (BMI); (4) complete blood count parameters, including white blood cell count (WBC), neutrophil count (NE), lymphocyte count (LY), monocyte count (Mo), and neutrophil-to-lymphocyte ratio (NLR); (5) inflammatory and biochemical parameters, including CRP, LDH, creatine kinase (CK), CK-MB, α-hydroxybutyrate dehydrogenase (α-HBDH), serum amyloid A (SAA), IL-6, erythrocyte sedimentation rate (ESR), and lactate (Lac); and (6) immune-related parameters, including anti-SARS-CoV-2 spike protein IgG, T-cell percentage (T-pct), absolute T-cell count (T-abs), CD8^+^ T-cell percentage (CD8-pct), absolute CD8^+^ T-cell count (CD8-abs), CD4^+^ T-cell percentage (CD4-pct), absolute CD4^+^ T-cell count (CD4-abs), CD4/CD8 ratio, NK-pct, absolute natural killer cell count (NK-abs), B-cell percentage (B-pct), and absolute B-cell count (B-abs). IL-6 was measured by electrochemiluminescence immunoassay on the Roche Cobas e801 platform (Roche Diagnostics, Mannheim, Germany), with a detection range of 1.5–5000 pg/mL. Vaccination status was recorded as a binary variable and was evaluated in an additional sensitivity analysis rather than included in the primary candidate-predictor set. Anti-SARS-CoV-2 spike protein IgG antibodies were measured using a magnetic-particle chemiluminescence immunoassay (Autobio Diagnostics Co., Ltd., Zhengzhou, China) on the AutoLumo A2000 Plus automated chemiluminescence immunoassay analyzer. The assay used in the present study measured anti-SARS-CoV-2 spike protein IgG antibodies rather than total serum IgG. The assay used recombinant SARS-CoV-2 spike protein antigen to detect spike-specific IgG antibodies, and the results were reported as the signal-to-cutoff ratio (S/CO), a dimensionless semiquantitative index. According to the manufacturer’s interpretation criteria, an S/CO value ≤0.79 was considered nonreactive, 0.80–1.20 was considered equivocal, and ≥1.21 was considered reactive. Routine calibration and internal quality-control procedures were performed in accordance with the manufacturer’s instructions and the laboratory’s standard operating procedures. The inter-assay coefficient of variation was <5%. Lymphocyte subsets were analyzed using a BD FACSCanto flow cytometer (BD Biosciences, San Jose, CA, USA) with EDTA-anticoagulated peripheral venous whole blood. Lymphocytes were identified on the basis of CD45 expression and low side scatter. For the BD Multitest 6-Color TBNK assay, the reagent panel comprised CD3-FITC (clone SK7), CD16-PE (clone B73.1), CD56-PE (clone NCAM16.2), CD45-PerCP-Cy5.5 (clone 2D1), CD4-PE-Cy7 (clone SK3), CD19-APC (clone SJ25C1), and CD8-APC-Cy7 (clone SK1). Within the gated lymphocyte population, CD3-negative cells were further classified according to combined CD16/CD56 and CD19 expression, with NK cells identified as CD3−CD19−CD16/56^+^ cells. NK-pct was calculated as the percentage of NK cells among gated lymphocytes. Specimens were processed and analyzed in accordance with the laboratory’s standardized operating procedures and the manufacturer’s instructions. Routine instrument performance checks and quality-control procedures were performed before sample acquisition, and gating was visually reviewed to ensure appropriate separation of lymphocyte subsets. The laboratory reference interval for NK-pct, established according to the laboratory SOP, was 5–27%. Time from symptom onset was incompletely documented in the retrospective records; therefore, time from symptom onset to admission could not be reliably calculated and was not included.

### 2.3. Sample Size Considerations and Cohort Allocation

This retrospective study consecutively enrolled all eligible patients treated during the predefined study period; therefore, the sample size was determined by the number of eligible patients available from January 2022 to December 2023 rather than by a prospective sample-size target. The 774 patients were randomly allocated at a 7:3 ratio to a training set (*n* = 543) for predictor selection and model development and a held-out internal validation set (*n* = 231) for internal performance evaluation. The training set contained 90 patients with severe/critical disease. After exclusion of the outcome and vaccination status, 34 candidate variables were considered; 24 variables were entered into the initial multivariable logistic regression, corresponding to an EPV of 90/24 = 3.75. This low early-stage EPV and the relatively large candidate-predictor set may reduce the stability of variable selection, even though consensus selection and cross-validation were used to mitigate overfitting. Accordingly, the present study should be regarded as single-center model development with internal evaluation, and larger multicenter cohorts are required for external validation.

### 2.4. Statistical Analysis

Among the 774 enrolled patients, the proportions of missing values for each variable are detailed in Table S1. Variables with >10% missing observations were excluded; for those with ≤10% missingness—including hypertension (2.94%), hyperlipidemia (3.87%), anti-SARS-CoV-2 spike protein IgG (9.75%), CRP (3.41%), LDH (1.55%), T-pct (3.72%), and B-abs (5.11%)—multiple imputation was performed using the random forest method (mice package, R). All 774 patients had complete outcome data and primary laboratory results, and no patient was excluded due to missing laboratory values. Continuous variables with a Gaussian distribution are presented as the mean ± SD and were compared using the independent-samples *t* test; skewed data are presented as the median (IQR) and were analyzed using the Mann–Whitney U test. Categorical variables are presented as *n* (%) and compared using the chi-square test or Fisher’s exact test. A two-tailed *p* < 0.05 was considered statistically significant. All analyses were performed in R (version 4.5.2) and Python (version 3.12).

### 2.5. Variable Screening Process

We implemented a consensus-based screening strategy combining three complementary approaches within the training set. First, variables showing *p* < 0.05 in univariable comparisons were entered into multivariable logistic regression with backward stepwise elimination. Second, LASSO regression was applied with the penalty parameter λ chosen by 10-fold cross-validation (λ_1se criterion), retaining predictors with non-zero coefficients. Third, for RFE-based selection, collinear variables (|r| ≥ 0.7 with FDR-adjusted *p* < 0.05) were identified from a correlation matrix and removed; the remaining candidates were ranked by a random forest classifier using five-fold cross-validation, with the AUC as the optimization criterion. Variables consistently identified by all three methods were carried forward as core predictors. Multicollinearity among the selected variables was assessed using the variance inflation factor (VIF), with values < 5 considered acceptable. To evaluate the stability of the RFE procedure, nested cross-validation (10 outer × 5 inner folds) was performed, and the coefficient of variation of feature-selection frequencies across folds was computed.

### 2.6. Model Construction and Evaluation

A multivariable logistic regression model incorporating the consensus predictors was fitted in the training set and represented as the CORIS nomogram. Discriminative ability was quantified by the area under the ROC curve (AUC), with 95% confidence intervals estimated from 1000 bootstrap resamples. At the optimal cutoff (Youden index), accuracy, sensitivity, specificity, positive predictive value, and negative predictive value were calculated. Agreement between predicted probabilities and observed outcomes was examined using calibration curves and the Hosmer–Lemeshow test (*p* > 0.05 denoting acceptable calibration); bootstrap correction (1000 resamples) was applied to the training-set calibration curve. Clinical net benefit was assessed by decision curve analysis across a spectrum of threshold probabilities. All model evaluation in the validation set used predicted probabilities generated exclusively by the model fitted in the training set.

### 2.7. Comparison with Previous Models

To benchmark the severity-discrimination performance of the CORIS nomogram against previously published models, two COVID-19 severity prediction models were selected: the lymphocyte subset-based nomogram developed by Li et al. [11] and the routine laboratory parameter-based model developed by Chen et al. [10]. Each model was refitted in our training cohort using its original predictor set. Because albumin was unavailable in the present dataset, CRP was substituted for albumin in the Chen model for the primary analysis; a reduced four-variable version (age, neutrophil count, LDH, lymphocyte count) was also examined as a sensitivity check. ROC curves were generated for all three models in the validation set, and AUC differences were evaluated with DeLong’s test. Decision curves were constructed to compare net clinical benefit across models. For the Li comparator, the model was reconstructed using the predictors shown in the final published nomogram: sex, age, absolute T-cell count, absolute B-cell count, and absolute helper T-cell count. The model was refitted in our training cohort and evaluated in the held-out internal validation set; this comparison therefore assessed the transportability of the published predictor set.

### 2.8. Sensitivity and Ablation Analyses

To examine the contribution of IL-6 and NK-pct, two ablated logistic models were fitted in the training set after removing IL-6 or NK-pct from the primary CORIS predictor set. Two additional sensitivity models added BMI or vaccination status separately to the five CORIS predictors. All models were evaluated in the held-out internal validation set. AUC differences relative to CORIS were tested using DeLong’s method, and Brier scores were calculated; adjusted odds ratios were reported for BMI and vaccination status.

## 3. Results

### 3.1. Baseline Characteristics of the Study Population

A total of 774 patients with SARS-CoV-2 infection who underwent complete laboratory testing and clinical evaluation were included in the study. According to disease severity, 646 patients were classified as having non-severe disease (83.46%), whereas 128 were classified as having severe or critical disease (16.54%). The median age of the overall study population was 41.0 years (IQR, 31.0–57.0), and 52.97% of the patients were male. The extent of missing data for each variable in the original dataset is summarized in Table S1.

Compared with patients in the non-severe group, those in the severe/critical group were significantly older (median age, 60.0 vs. 39.0 years; *p* < 0.001) and more likely to be male (68.75% vs. 49.85%; *p* < 0.001). The prevalence of hypertension (76.56% vs. 56.19%; *p* < 0.001) and the proportion of patients with a history of smoking (32.03% vs. 19.97%; *p* = 0.004) were also significantly higher in the severe/critical group. By contrast, no significant between-group differences were observed in the prevalence of diabetes mellitus or hyperlipidemia (both *p* > 0.05). The overall prevalences of diabetes mellitus, hypertension, and hyperlipidemia were 53.75%, 59.56%, and 50.39%, respectively.

Patients with severe/critical COVID-19 exhibited a generally less favorable inflammatory and immunological profile. With respect to inflammatory and biochemical markers, NLR, Mo, CRP, LDH, SAA, IL-6, and ESR were all significantly higher in the severe/critical group than in the non-severe group (*p* < 0.001). Regarding immune cell subsets, patients in the severe/critical group had significantly lower lymphocyte counts, higher NK-cell percentages and absolute counts, and lower B-cell percentages (*p* < 0.05). Significant differences were also observed in several biochemical and immune-related indicators, with higher anti-SARS-CoV-2 spike protein IgG S/CO values and α-hydroxybutyrate dehydrogenase levels but lower creatine kinase levels in the severe/critical group (*p* < 0.01). In addition, body mass index and T-cell subset-related parameters, including the CD4/CD8 ratio and CD8^+^ T-cell percentage, differed significantly between the two groups (*p* < 0.05). Detailed baseline characteristics are presented in Table 1.

To ensure the reliability of model development and validation, the overall cohort was randomly divided at a 7:3 ratio into a training cohort (*n* = 543) and a validation cohort (*n* = 231). The proportions of patients with severe/critical COVID-19 in the training and validation cohorts were 16.57% and 16.45%, respectively, which were comparable to the proportion in the overall cohort (16.54%). No statistically significant differences were identified between the training and validation cohorts in sex distribution, age, prevalence of underlying diseases, or any of the inflammatory, biochemical, and immune-related baseline variables (*p* > 0.05), indicating that the random allocation yielded comparable cohorts. After stratification by disease severity within the training and validation cohorts, the baseline characteristics of the respective subgroups were consistent with those observed in the overall non-severe and severe/critical groups. In both cohorts, severe/critical disease was characterized by elevated inflammatory markers and altered distributions of immune cell subsets. Detailed baseline characteristics of the training and validation cohorts are provided in Tables S2 and S3, respectively, and direct comparisons between the two cohorts are presented in Table S4.

### 3.2. Feature Selection

To develop a robust and interpretable discrimination model, a systematic multistep feature-selection procedure was implemented using the training cohort (*n* = 543). Three complementary approaches—multivariable logistic regression, RFE, and LASSO regression—were applied, and variables consistently identified across all three methods were retained as the final core predictors. First, univariable analysis was performed for 34 variables from the overall baseline table (Table 1), excluding the outcome indicator and vaccination status. The 24 variables with *p* < 0.05 were entered into multivariable logistic regression (the 10 variables excluded for having *p* ≥ 0.05 were: alcohol consumption, diabetes, hyperlipidemia, Lac, CK-MB, T-pct, CD8-abs, CD4-pct, CD4-abs, and B-abs). Following backward stepwise selection in multivariable logistic regression, nine independent predictors were retained: age, IgG, LDH, IL-6, CD8-pct, NK-pct, NK-abs, B-pct, and Mo. The results of the multivariable logistic regression analysis are presented in Table 2. The 15 excluded variables were sex, smoking, hypertension, BMI, WBC, NE, LY, NLR, CRP, CK, HD, SAA, ESR, T-abs, and CD4-CD8 ratio, all of which had *p* ≥ 0.05 in the multivariable model.

In the RFE analysis, the 31 candidate variables remaining after collinearity assessment were first ranked, and five-fold cross-validation was performed using a random forest classifier. In Figure 2A, the bars indicate the normalized random forest feature importance of each variable and correspond to the left y-axis; the line represents the mean cross-validation AUC obtained as variables were added sequentially according to the RFE ranking and corresponds to the right y-axis; and the shaded band represents the 95% CI of the five-fold cross-validation AUC. The first eight variables and their corresponding AUC line are highlighted in red. The mean AUC increased as the number of variables increased and reached a relatively stable plateau after eight variables were included; adding further variables did not yield an appreciable improvement in discrimination. Therefore, RFE retained eight variables: IgG, LDH, age, IL-6, NK-pct, NLR, CRP, and CK.

Meanwhile, LASSO regression was applied to all 34 candidate variables. Figure 2B presents the ten-fold cross-validation error curve used to select the penalty parameter λ. The x-axis represents log(λ), and the y-axis represents mean binomial deviance; red points indicate the mean cross-validation error at each λ value, and gray error bars indicate one standard error. The two vertical dashed lines denote λ_min, which minimized the cross-validation error, and λ_1se, which was selected according to the one-standard-error rule. We selected λ_1se, shown by the right dashed line (λ_1se = 0.03755855), to obtain a more parsimonious predictor set.

Figure 2C shows the LASSO coefficient paths for the 34 candidate variables. Each colored curve represents the regression coefficient of one variable; the x-axis represents log(λ), and the y-axis represents the corresponding regression coefficient. As λ increased, the strength of penalization increased and the coefficients progressively shrank toward zero. The numbers along the top indicate the number of variables with nonzero coefficients at each λ value. At λ_1se, nine variables retained nonzero coefficients: age, IgG, NLR, CRP, LDH, IL-6, ESR, NK-pct, and Mo; the coefficients of the remaining 25 variables were shrunk to zero.

Figure 2 presents the variable-selection processes of RFE and LASSO and does not, by itself, determine the final model predictors. RFE retained eight variables, LASSO retained nine variables, and multivariable logistic regression retained nine variables. The intersection of the predictors selected by the three methods was then taken, yielding five consistently selected variables: age, LDH, IgG, IL-6, and NK-pct. These five variables were used to construct the CORIS nomogram. The complete feature-selection and model-development pipeline is detailed in Figure S2. Variance inflation factor analysis showed that all five variables had VIF values below 5, indicating no substantial multicollinearity (Table S5). Collectively, these predictors represented several major pathophysiological dimensions, including demographic susceptibility represented by age, tissue injury and metabolic disturbance represented by LDH, systemic inflammation represented by IL-6, and humoral and cellular immune responses represented by IgG and NK-pct. Their overlap with the variables that differed significantly between the severe/critical and non-severe groups further supported the biological plausibility and clinical interpretability of the selected feature set.

The stability of the RFE procedure was further evaluated using nested cross-validation with 10 outer folds and 5 inner folds. RFE was independently performed within each fold to select the eight highest-ranking features. The AUCs across the 10 outer test folds ranged from 0.897 to 0.915, indicating limited variation in model performance. Age, IgG, CRP, LDH, and NK-pct were selected in all 10 folds, IL-6 was selected in 9 folds, and NLR and CK were selected in 8 folds (Table S6). The coefficient of variation for feature-selection frequency was 0.195, indicating good stability of the RFE procedure. These findings suggest that the selection of the principal predictors was not dependent on a specific data partition. Variation among the lower-ranked features further supported the use of consensus selection across the three complementary methods to determine the final predictor set.

### 3.3. Nomogram Development and Model Evaluation

A multivariable logistic regression model incorporating the five core predictors was fitted in the training set; the regression coefficients, odds ratios (ORs), and 95% confidence intervals (CIs) are presented in Table S7. The results showed that each one-unit increase in NK-pct was associated with a 6.9% increase in the odds of being classified as severe/critical at admission (OR = 1.069, 95% CI: 1.041–1.099, *p* < 0.001); each one-year increase in age was associated with a 4.8% increase in the odds of being classified as severe/critical at admission (OR = 1.048, 95% CI: 1.028–1.069, *p* < 0.001); and each one-unit increase in LDH, IgG, and IL-6 was associated with a 0.9% (OR = 1.009), 0.6% (OR = 1.006), and 0.8% (OR = 1.008) increase in the odds of severe/critical classification at admission, respectively, all of which were statistically significant (*p* < 0.05) (Table S7). Based on the five core predictors identified above—age, LDH, IgG, IL-6, and NK-pct—a multivariable logistic regression model was fitted in the training cohort (*n* = 543), and a corresponding nomogram was constructed. Because the model integrates routine laboratory parameters with immune-related indicators, it was designated the CORIS nomogram and was developed to enable individualized discrimination between non-severe and severe/critical COVID-19 (Figure 3A). To illustrate the clinical application workflow of the CORIS nomogram more clearly, one representative low-risk patient and one representative high-risk patient were selected for nomogram-based demonstrations. Detailed information is provided in the Supplementary Materials (Clinical Case Demonstration). The full model was logit(*p*) = −8.317 + 0.047 × Age + 0.009 × LDH + 0.006 × IgG + 0.008 × IL-6 + 0.067 × NK-pct, where *p* = 1/[1 + exp(−logit(*p*))] is the estimated probability of severe/critical status at admission. Age was entered in years, LDH in U/L, anti-SARS-CoV-2 spike protein IgG as the dimensionless S/CO index, IL-6 in pg/mL, and NK-pct as a percentage. The validation-set Youden-optimal cutoff reported for descriptive classification performance was 0.238 and requires prospective evaluation before use as a fixed clinical decision threshold.

In the training cohort, the CORIS nomogram demonstrated strong discriminative performance, with a bootstrap-corrected AUC of 0.914 (95% CI, 0.884–0.940) based on 1000 bootstrap resamples (Figure S3A). The bootstrap-corrected calibration curve closely approximated the ideal reference line (Figure S3B), and the Hosmer–Lemeshow goodness-of-fit test indicated satisfactory calibration (χ^2^ = 9.2428, df = 8, *p* = 0.3222). The calibration intercept was 0.000 (95% CI, −0.306 to 0.306), the calibration slope was 1.000 (95% CI, 0.812 to 1.188), and the Brier score was 0.079. Decision curve analysis further showed that the CORIS nomogram yielded a positive clinical net benefit across threshold probabilities ranging from 0 to approximately 0.95 (Figure S3C). Moreover, the nomogram achieved a significantly higher AUC than any individual predictor, as detailed in Table 3.

When the model developed in the training cohort was applied to the validation cohort (*n* = 231), it retained excellent discriminative performance, with an AUC of 0.918 (95% CI, 0.868–0.960); the confidence interval was estimated using 1000 bootstrap resamples of the validation data (Figure 3B). The apparent calibration curve showed close agreement between the predicted and observed probabilities (Figure 3C), which was further supported by the Hosmer–Lemeshow test (χ^2^ = 5.894, df = 8, *p* = 0.6591). The calibration intercept was -0.406 (95% CI, -0.887 to 0.076), the calibration slope was 0.997 (95% CI, 0.714 to 1.279), and the Brier score was 0.074, further supporting good model calibration. Decision curve analysis demonstrated a consistently positive clinical net benefit across threshold probabilities ranging from 0 to approximately 0.80 (Figure 3D). Overall, the held-out results were consistent with those obtained in the training cohort and support favorable internal performance; they do not constitute external validation. A consolidated summary of discrimination and calibration, including 95% confidence intervals for the calibration intercept and slope, is provided in Table S8.

### 3.4. Comparison of the CORIS Nomogram with Previous Models

To further benchmark the severity-discrimination performance of the CORIS nomogram, the model developed in the training cohort (*n* = 543) was applied to the validation cohort (*n* = 231) and compared with two previously published COVID-19 severity models. The nomogram developed by Li et al. [11] included sex, age, absolute T-cell count (T-abs), absolute B-cell count (B-abs), and absolute helper T-cell count; the latter corresponded to absolute CD4^+^ T-cell count (CD4-abs) in the present dataset. The model reported by Chen et al. [10] incorporated five routine laboratory variables: age, NE, LDH, LY, and albumin (ALB). Because ALB was unavailable in the present dataset, a five-variable modified version of the Chen model, in which CRP was substituted for ALB, was used in the primary analysis. In addition, a reduced four-variable version comprising the available original predictors—age, NE, LDH, and LY—was evaluated in a sensitivity analysis to determine whether the substitution materially affected the comparative findings. Both the Li and Chen models were refitted in the present training cohort using their respective predictor sets and subsequently evaluated against the CORIS nomogram in the validation cohort.

As shown in the comparative receiver operating characteristic curves in Figure 4A, the CORIS nomogram achieved an AUC of 0.918 (95% CI, 0.868–0.960), significantly exceeding that of the corrected Li 2021 comparator (AUC, 0.811; 95% CI, 0.745–0.872; AUC difference, 0.107; 95% CI, 0.045–0.169; DeLong *p* < 0.001) and the modified Chen model (AUC, 0.839; 95% CI, 0.768–0.901; DeLong *p* = 0.003). In the sensitivity analysis, removal of CRP and refitting of the reduced four-variable Chen model produced no change in the validation AUC. Moreover, the predicted probabilities generated by the four- and five-variable versions were highly correlated (Spearman’s ρ = 0.995), indicating that CRP provided little additional discriminative information beyond age, NE, LDH, and LY.

At the optimal cutoff, the CORIS nomogram yielded an accuracy of 0.870, a sensitivity of 0.842, and a specificity of 0.876, outperforming both comparator models across these performance metrics (Table 4). Decision curve analysis further demonstrated that the CORIS nomogram provided a greater net clinical benefit than the Li and Chen models across a broader range of threshold probabilities (Figure 4B), supporting its greater potential clinical utility.

### 3.5. Sensitivity and Ablation Analyses

In the internal validation set, removing IL-6 reduced the AUC from 0.918 to 0.907 (95% CI, 0.856–0.950; DeLong *p* = 0.018), whereas removing NK-pct reduced the AUC to 0.885 (95% CI, 0.814–0.945; DeLong *p* = 0.013), supporting the incremental contribution of both immune-related predictors (Table 4).

Adding BMI produced an AUC of 0.919 (95% CI, 0.868–0.961; ΔAUC = +0.001; DeLong *p* = 0.161), and BMI was not independently associated with admission severity status (adjusted OR, 0.993; 95% CI, 0.923–1.069; *p* = 0.855). Adding vaccination status produced an AUC of 0.920 (95% CI, 0.868–0.962; ΔAUC = +0.002; DeLong *p* = 0.291), and vaccination status was not independently associated with admission severity status (adjusted OR, 0.687; 95% CI, 0.255–1.847; *p* = 0.457). Thus, neither addition materially changed model discrimination in this cohort (Table S9).

## 4. Discussion

In this study, clinical data from 774 patients with COVID-19 were analyzed to systematically characterize differences between patients with non-severe disease and those with severe or critical disease across multiple domains, including demographic characteristics, inflammatory markers, biochemical parameters, and immune cell subsets. Based on these findings, we developed a novel CORIS nomogram. Using three complementary feature-selection approaches, nested cross-validation, and evaluation in a held-out internal validation set, the CORIS nomogram demonstrated favorable discriminatory performance in distinguishing patients with non-severe COVID-19 from those with severe or critical disease. Diabetes was numerically less frequent in the severe/critical group than in the non-severe group, but the difference was not statistically significant (*p* = 0.522). This finding should not be interpreted as an inverse or protective association.

For predictor selection, multivariable logistic regression, RFE, and LASSO regression were applied, and their intersection was used to identify five core predictors: age, LDH, anti-SARS-CoV-2 spike protein IgG, IL-6, and NK-pct [16,17]. Consensus selection, rather than inclusion of the union of all selected variables, was used to impose a stringent selection criterion and reduce the number of predictors, thereby improving model parsimony while preserving representation of clinically relevant domains. All five predictors were consistently identified by the three methods. Moreover, nested cross-validation further demonstrated that age, anti-SARS-CoV-2 spike protein IgG, LDH, and NK-pct were selected in all 10 outer folds, whereas IL-6 was selected in 9 of 10 folds, supporting the stability of the feature-selection process [18]. All variance inflation factor values were below 5, indicating no substantial multicollinearity among the selected predictors [19]. The resulting predictor set represented four major clinical and pathophysiological dimensions: demographic susceptibility, reflected by age; tissue injury, reflected by LDH; systemic inflammation, reflected by IL-6; and humoral and cellular immune status, reflected by anti-SARS-CoV-2 spike protein IgG and NK-pct, respectively. This multidimensional integration has been infrequently incorporated into previously reported COVID-19 nomograms [6,20]. Collectively, the three methods identified 13 unique candidate predictors: 9 were selected by multivariable logistic regression, 8 by RFE, and 9 by LASSO regression. Including the full union of these candidate predictors would have increased model complexity and potentially introduced redundant information. In contrast, consensus selection reduced the final model to five predictors while retaining the most reproducible signals identified across all three approaches, thereby balancing discriminatory performance with model parsimony. In addition, these predictors can be measured using established clinical laboratory procedures, which may facilitate implementation of the CORIS nomogram across healthcare settings with different resource levels.

The CORIS nomogram demonstrated excellent discrimination, with an AUC of 0.914 (95% CI, 0.884–0.940) in the training cohort and 0.918 (95% CI, 0.868–0.960) in the validation cohort, exceeding the discriminatory performance of each individual predictor. The Hosmer–Lemeshow goodness-of-fit test yielded *p* values of 0.3222 and 0.6591 in the training and validation cohorts, respectively, and the calibration curves closely approximated the ideal reference line, indicating satisfactory agreement between predicted and observed probabilities [21,22]. Decision curve analysis further demonstrated that the CORIS nomogram provided a positive net benefit across threshold probabilities of approximately 0–0.95 in the training cohort and 0–0.80 in the validation cohort [23]. Overall, these findings support the potential clinical utility of the model for individualized assessment of COVID-19 severity.

From a pathophysiological perspective, the five CORIS predictors reflect distinct but interconnected processes underlying severe COVID-19. Age is a well-established risk factor for severe disease, potentially reflecting immunosenescence and a greater burden of comorbidities in older adults [24]. LDH is released following cellular injury, and elevated circulating levels may indicate extensive tissue damage and multiorgan involvement [25,26,27]. Consistent with its prognostic relevance, LDH is also included in established COVID-19 risk models, such as the CALL score and COVID-GRAM [28,29]. IL-6 mediates systemic hyperinflammation and immune dysregulation, and elevated levels are associated with greater disease severity and poorer outcomes [30,31,32]. The anti-SARS-CoV-2 spike protein IgG S/CO index was higher in patients with severe or critical disease, but this semi-quantitative signal may reflect a combination of disease severity, prior vaccination or infection, and sampling time [33,34,35]. The higher NK-pct in patients with severe or critical disease warrants particular consideration. Although NK-abs was also elevated, NK-pct was retained by all three feature-selection methods, whereas NK-abs was selected only by multivariable logistic regression and excluded by LASSO regression and RFE [36,37,38]. In the present cohort, NK-pct may therefore capture both changes in NK-cell abundance and relative expansion of the NK-cell compartment within a lymphocyte pool affected by T- and B-cell depletion or redistribution, thereby reflecting broader disruption of lymphocyte composition. By integrating NK-cell abundance with shifts in overall lymphocyte composition, NK-pct may provide greater discriminatory information than NK-abs, which may explain its retention in the final five-variable model.

The five CORIS predictors represent complementary clinical domains related to COVID-19 severity, including inflammation, immune-cell composition, tissue injury, and host susceptibility [39,40,41]. These domains have been discussed in previous studies of severe COVID-19, but that literature provides biological context rather than direct mechanistic evidence from the present cohort [41]. IL-6, anti-SARS-CoV-2 spike protein IgG, NK-pct, LDH, and age may therefore provide nonredundant information for admission severity discrimination; however, the present retrospective analysis cannot establish a causal immunopathological pathway among these markers. Previous studies have reported associations between Fc glycosylation patterns of anti-SARS-CoV-2 spike protein IgG and inflammatory responses in severe COVID-19 [42]. Age-related immune changes may also contribute to differences in disease severity [43]. Compared with previous models, Li et al. (2021) combined sex and age with absolute T-cell, B-cell, and helper T-cell counts, whereas Chen et al. (2024) emphasized routine laboratory parameters; neither integrated the same combination of immune, inflammatory, and tissue-injury indicators used in CORIS. The CORIS variables should therefore be interpreted as a pragmatic multivariable marker set for admission severity discrimination rather than as a mechanistic framework or a basis for selecting immunomodulatory treatment [44].

To further evaluate its severity-discrimination performance, the CORIS nomogram was compared in the validation cohort with two previously published COVID-19 severity models developed by Li and Chen [10,11]. The CORIS nomogram achieved an AUC of 0.918, exceeding that of the Li comparator (AUC, 0.811; DeLong *p* < 0.001) and the modified Chen model (AUC, 0.839; DeLong *p* = 0.003). It also showed improvements in accuracy, sensitivity, and specificity. The favorable performance of the CORIS nomogram may be attributable to its integration of complementary inflammatory and immunological dimensions, together with a consensus-selected predictor set characterized by low redundancy and high clinical interpretability. It should be noted that the original Chen model included albumin, which was unavailable in the present dataset and was therefore replaced by CRP. Although albumin and CRP are not interchangeable biomarkers, both are closely related to systemic inflammatory status, and hypoalbuminemia and elevated CRP frequently coexist in patients with severe COVID-19. Thus, the substitution had some clinical rationale but may nevertheless have influenced the comparative results. In the sensitivity analysis, a reduced Chen model was constructed using the four available original predictors—age, neutrophil count, LDH, and lymphocyte count. Removal of CRP did not change the validation AUC, and the CORIS nomogram remained superior to the reduced model (DeLong *p* = 0.003), suggesting that substitution of CRP for albumin did not alter the overall direction of the comparison. Nevertheless, because these comparisons were conducted using a single-center validation cohort, the apparent superiority of the CORIS nomogram requires confirmation in independent external populations [21,45,46].

The comparison also provided insight into the transportability of predictor sets developed during different phases of the pandemic. The Li model was developed in Wuhan during the circulation of the ancestral SARS-CoV-2 strain in 2020 and achieved a C-index of 0.852 in its original validation cohort, whereas the present cohort was recruited during a period predominantly characterized by Omicron-lineage circulation [47]. After refitting the published predictor set of sex, age, T-abs, B-abs, and CD4-abs, the Li comparator achieved an AUC of 0.811 (95% CI, 0.745–0.872) in the present internal validation cohort. This finding indicates that the predictor set retained meaningful discriminative ability, although its performance was lower than that of CORIS. Differences between the original and present estimates should be interpreted cautiously because of differences in cohort composition, outcome distribution, laboratory measurement, and model fitting. Variant-related clinical and immunological differences may also have contributed to the observed performance difference [48,49]. These factors complicate direct cross-cohort comparisons. In contrast, the Chen model was developed during the Omicron-dominant period in 2022–2023 [10]. Its original validation AUC was 0.758, compared with 0.839 in the present validation cohort, suggesting that routinely measured predictors such as age and LDH may be relatively robust across populations and epidemic periods. The heterogeneous performance of the three models across different populations and variant backgrounds highlights the need for periodic updating and external validation of COVID-19 severity models as the virus evolves [20].

Several limitations of this study should also be acknowledged. First, this was a retrospective, single-center study conducted in an infectious disease hospital, which may have introduced selection bias. Patient demographic characteristics, comorbidity profiles, treatment strategies, and admission criteria may differ across healthcare institutions. These factors can lead to inter-institutional variability in the distribution and effect sizes of predictors, thereby affecting model discrimination and calibration. Consequently, the performance of the model in populations from other institutions requires further validation through multicenter external cohorts. Second, the training set included only 90 severe cases, and both feature selection and model construction were performed using the same training set, posing a residual risk of overfitting. The events-per-variable ratio (EPV) during the initial screening phase was only 3.75; although this was mitigated by consensus selection across three methods and the use of cross-validation, the combination of a large number of candidate variables with a relatively small number of events may still have affected the stability of variable selection, warranting further validation in external cohorts with larger sample sizes. Third, IL-6 measurement lacks uniform standardization across laboratories, and differences in sensitivity, linear range, and reference intervals among assay platforms may affect the transportability of cutoff values. This issue should be evaluated in future multicenter validation studies. Fourth, variables were measured only once within 24 h of admission, precluding assessment of biomarker trajectories. Longitudinal studies are needed to determine whether serial measurements provide incremental predictive value. Moreover, time from symptom onset to admission was unavailable, limiting interpretation of time-dependent markers such as anti-SARS-CoV-2 spike protein IgG, IL-6, LDH, and lymphocyte-subset measurements. Future prospective studies should incorporate symptom-onset timing and serial biomarker measurements and may further evaluate endothelial-injury or thromboinflammatory biomarkers, including sCLEC-2 and soluble thrombomodulin (sTM). The present model addresses admission severity discrimination only and should not be interpreted as predicting survival, subsequent deterioration, or thrombosis.

## 5. Conclusions

This study identified age, LDH, anti-SARS-CoV-2 spike protein IgG, IL-6, and NK-pct as independent predictors for distinguishing non-severe from severe/critical COVID-19. The CORIS nomogram, developed using these variables, integrates routine laboratory parameters with immune-related indicators across multiple pathophysiological dimensions and demonstrated favorable discrimination, calibration, and clinical net benefit. It may therefore provide an objective and practical tool for admission severity assessment and clinical triage among adults hospitalized with laboratory-confirmed COVID-19. Although the model outperformed both the lymphocyte subset-based model and the routine laboratory parameter-based model in the single-center validation set, its generalizability and comparative advantage require further confirmation in independent, multicenter external cohorts. The CORIS nomogram is intended for admission severity discrimination; it is not intended to diagnose SARS-CoV-2 infection or predict prognosis or post-admission events.

## Figures and Tables

**Figure 1:**
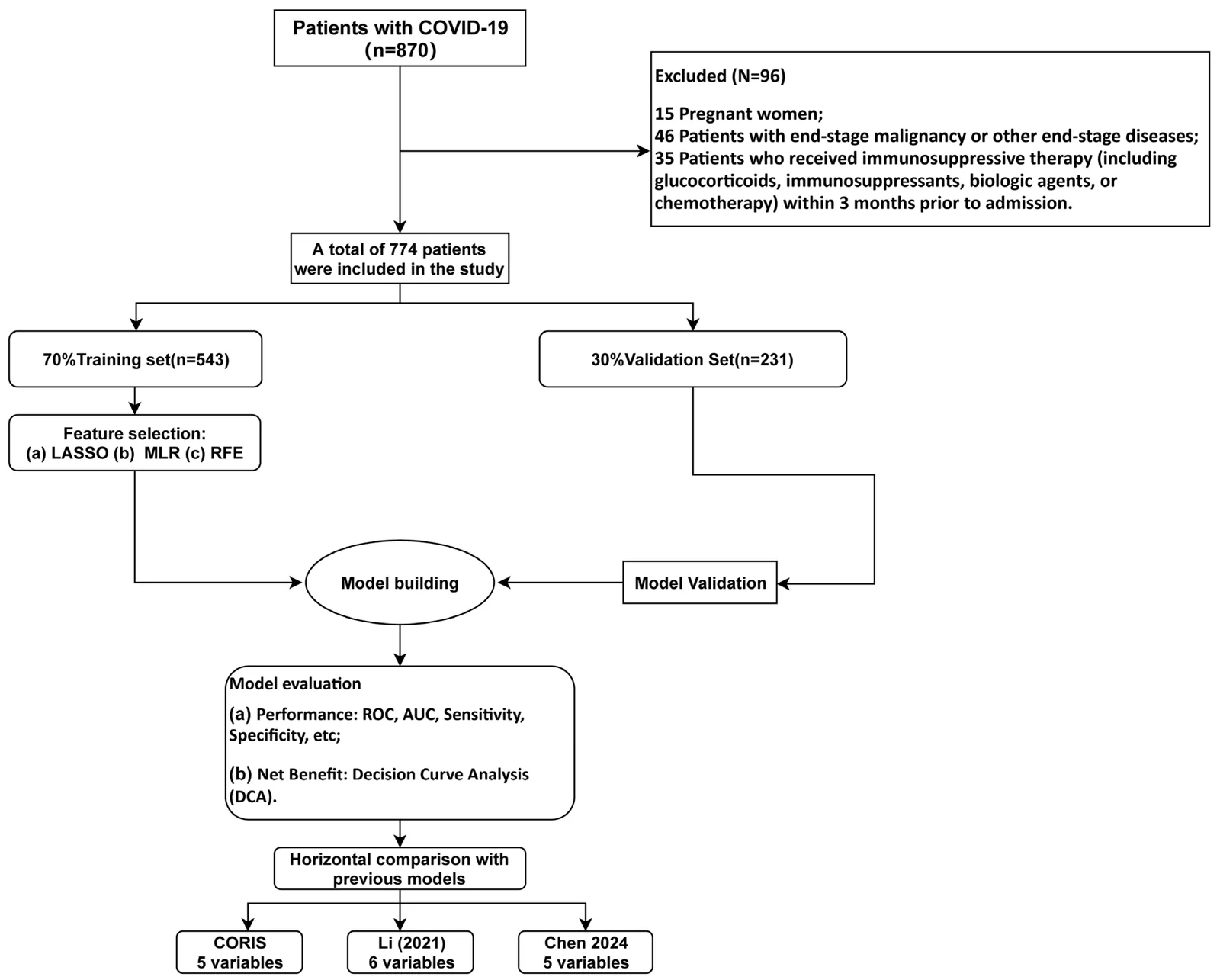
Study design and analysis workflow [10,11]. Abbreviations: RFE, recursive feature elimination; LASSO, least absolute shrinkage and selection operator; CORIS, COVID Routine-Immune Score nomogram; ROC, receiver operating characteristic; AUC, area under the ROC curve; DCA, decision curve analysis; VIF, variance inflation factor.

**Figure 2:**
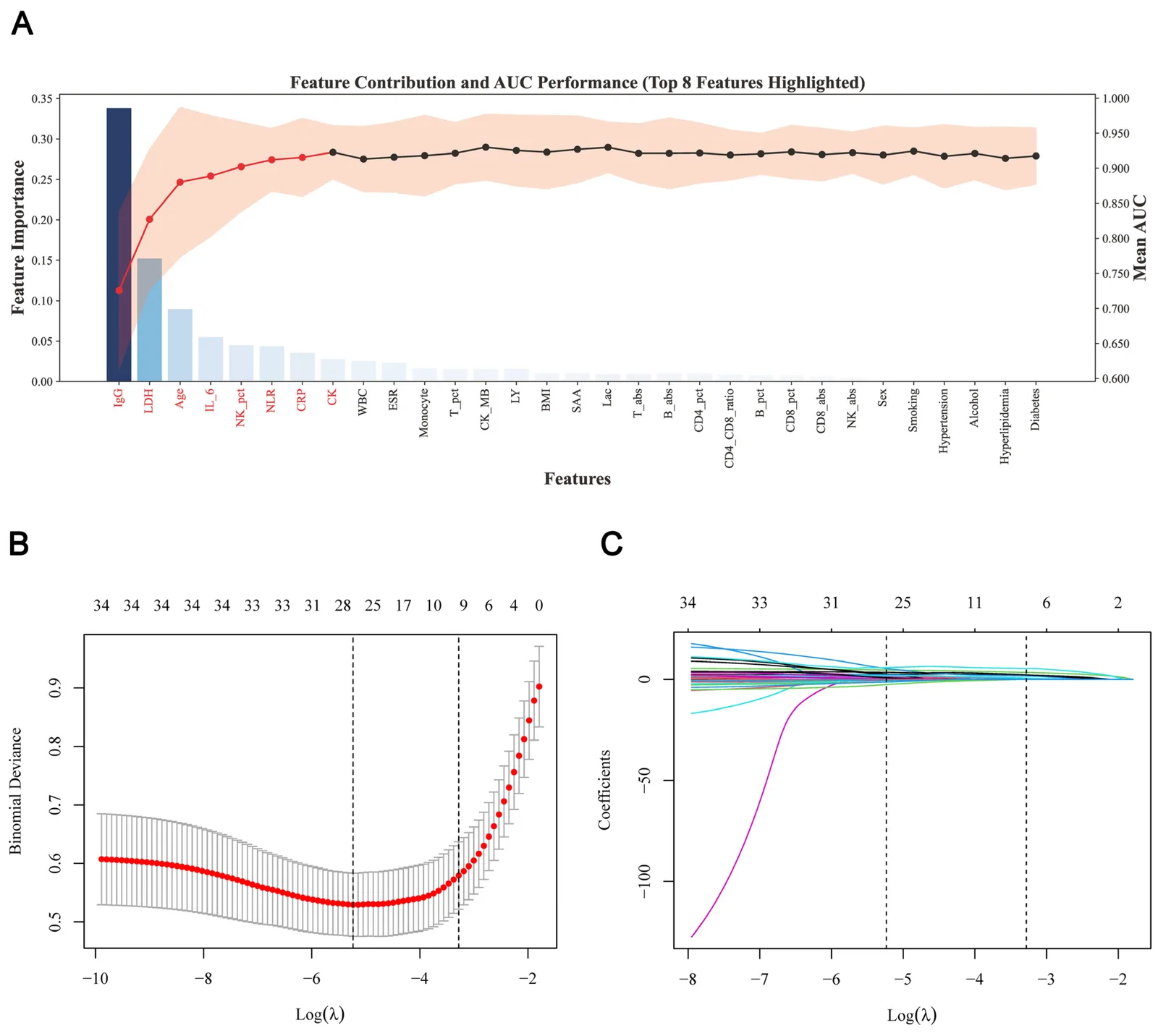
Feature selection by recursive feature elimination and LASSO regression. (**A**) Random forest-based RFE analysis. Bars indicate normalized feature importance, while the line and shaded band represent the mean five-fold cross-validation AUC and its 95% CI. The eight variables highlighted in red were retained. (**B**) Ten-fold cross-validation curve for LASSO. The dashed lines indicate λ_min and λ_1se; λ_1se was used for variable selection. (**C**) LASSO coefficient paths. Each curve represents the coefficient of one candidate variable. Nine variables with non-zero coefficients were retained at λ_1se. Abbreviations: RFE, recursive feature elimination; LASSO, least absolute shrinkage and selection operator; BMI, body mass index; IgG, anti-SARS-CoV-2 spike protein immunoglobulin G reported as dimensionless S/CO; WBC, white blood cell count; NE, neutrophil count; LY, lymphocyte count; NLR, neutrophil-to-lymphocyte ratio; Lac, lactate; CRP, C-reactive protein; LDH, lactate dehydrogenase; CK, creatine kinase; CK-MB, creatine kinase isoenzyme MB; HD, α-hydroxybutyrate dehydrogenase; SAA, serum amyloid A; IL-6, interleukin-6; ESR, erythrocyte sedimentation rate; T-pct, T cell percentage; T-abs, T cell absolute count; CD8-pct, CD8^+^ T cell percentage; CD8-abs, CD8^+^ T cell absolute count; CD4-pct, CD4^+^ T cell percentage; CD4-abs, CD4^+^ T cell absolute count; NK-pct, NK cell percentage; NK-abs, NK cell absolute count; B-pct, B cell percentage; B-abs, B cell absolute count.

**Figure 3:**
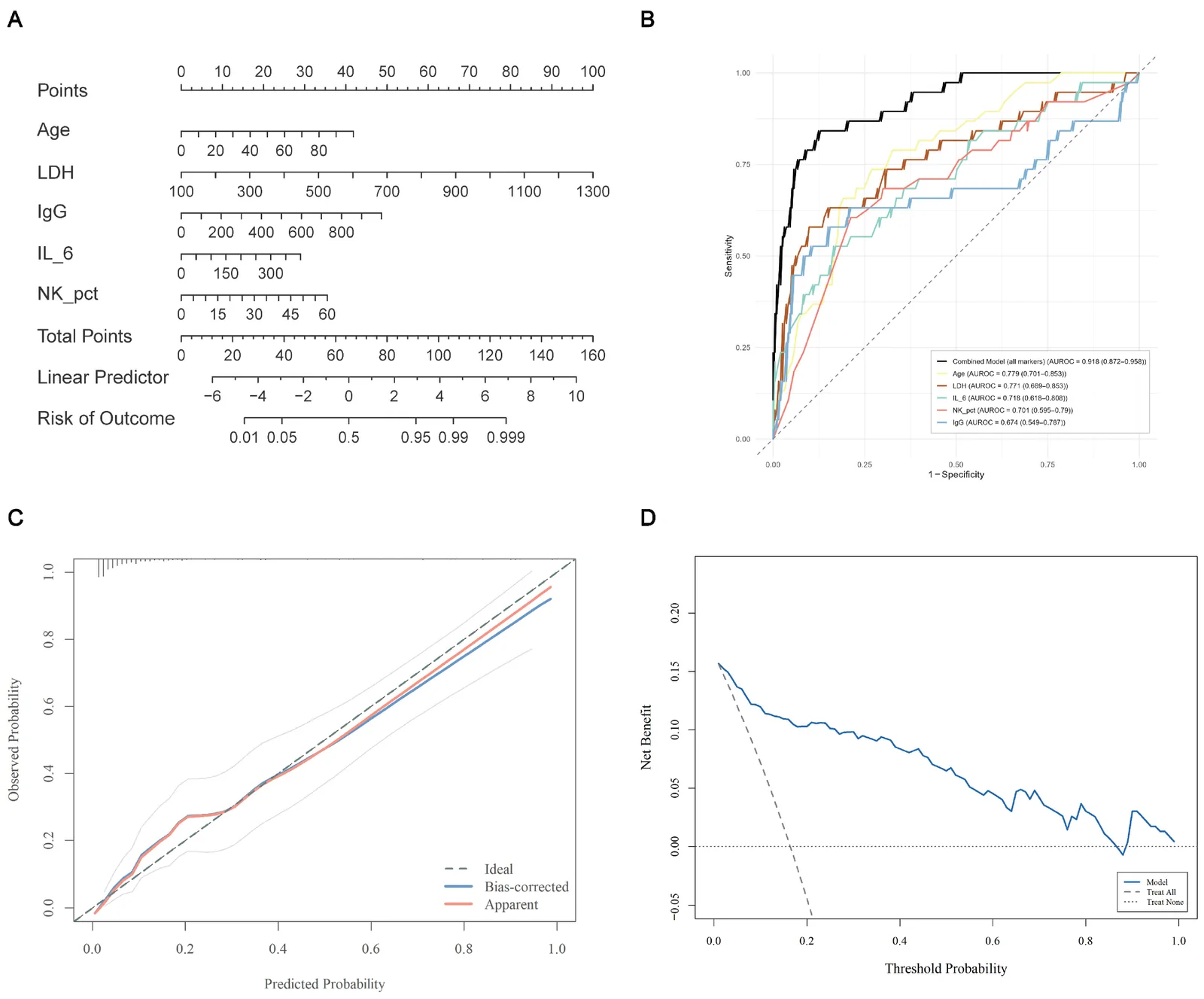
The CORIS nomogram and its performance evaluation in the validation set. (**A**) CORIS nomogram for differentiating severe/critical from non-severe COVID–19. (**B**) ROC curve (AUC = 0.918, 95% CI by bootstrap, 1000 iterations). (**C**) Calibration curve (H–L test: χ^2^ = 5.894, df = 8, *p* = 0.6591). (**D**) Decision curve analysis. Abbreviations: CORIS, COVID Routine-Immune Score nomogram; ROC, receiver operating characteristic; AUC, area under the ROC curve; DCA, decision curve analysis; LDH, lactate dehydrogenase; IgG, anti-SARS-CoV-2 spike protein immunoglobulin G reported as dimensionless S/CO; IL-6, interleukin-6; NK-pct, NK cell percentage.

**Figure 4:**
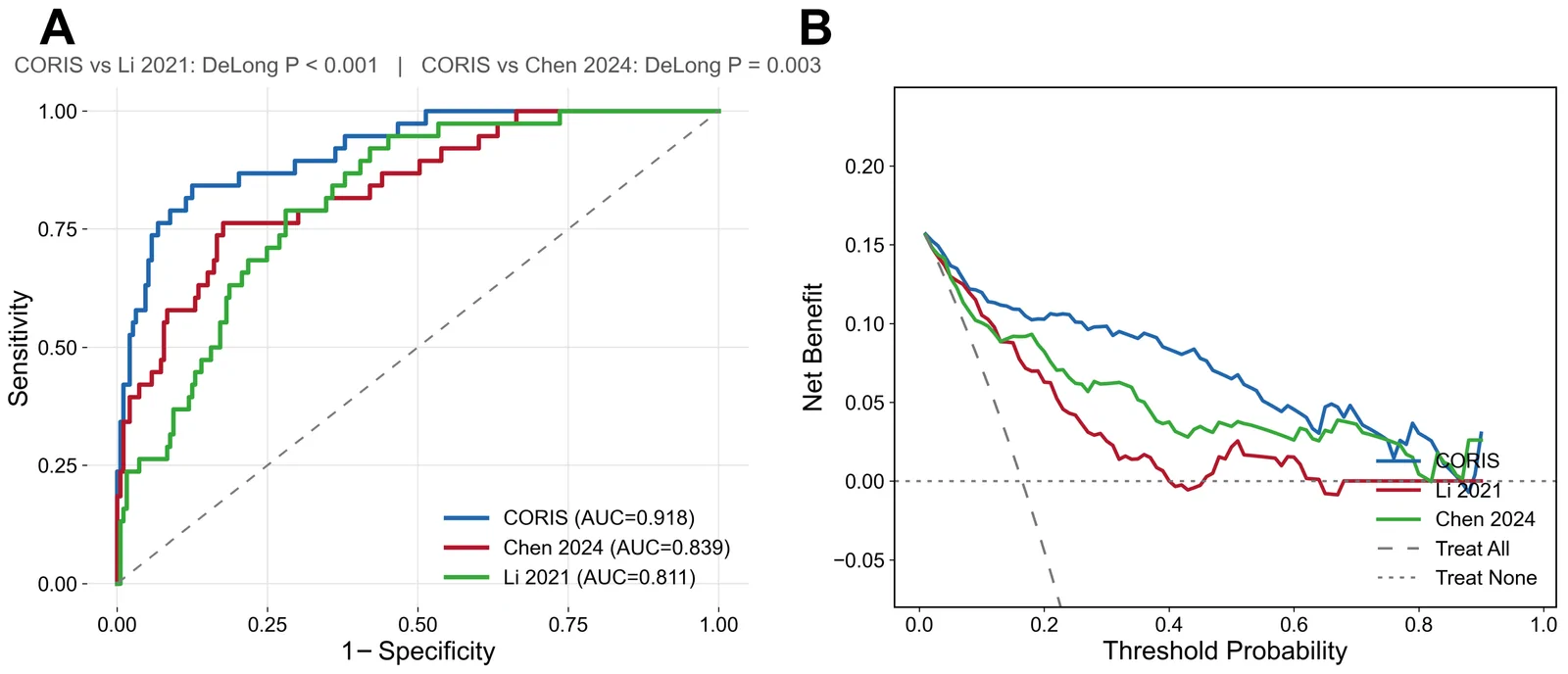
Comparison of the CORIS nomogram with previously published models in the validation set. (**A**) ROC curves of CORIS, Li 2021, and Chen 2024 models [10,11]. All models were refitted on the training set before evaluation. (**B**) Decision curve analysis of the three models. Abbreviations: CORIS, COVID Routine-Immune Score nomogram; ROC, receiver operating characteristic; AUC, area under the ROC curve; DCA, decision curve analysis.

**Table 1:** Baseline characteristics of the overall study population.

Variables	Total (*n* = 774)	Non-Severe (*n* = 646)	Severe (*n* = 128)	*p*
Vaccination status, *n* (%)				0.097
Yes	709 (91.60)	597 (92.41)	112 (87.50)	
No	65 (8.40)	49 (7.59)	16 (12.50)	
Sex				<0.001
Male	410 (52.97)	322 (49.85)	88 (68.75)	
Female	364 (47.03)	324 (50.15)	40 (31.25)	
Smoking				0.004
Yes	170 (21.96)	129 (19.97)	41 (32.03)	
No	604 (78.04)	517 (80.03)	87 (67.97)	
Alcohol				0.254
Yes	190 (24.55)	153 (23.68)	37 (28.91)	
No	584 (75.45)	493 (76.32)	91 (71.09)	
Diabetes				0.522
Yes	416 (53.75)	351 (54.33)	65 (50.78)	
No	358 (46.25)	295 (45.67)	63 (49.22)	
Hypertension				<0.001
Yes	461 (59.56)	363 (56.19)	98 (76.56)	
No	313 (40.44)	283 (43.81)	30 (23.44)	
Hyperlipidemia				0.846
Yes	390 (50.39)	324 (50.15)	66 (51.56)	
No	384 (49.61)	322 (49.85)	62 (48.44)	
Age	41.00 [31.00, 57.00]	39.00 [29.00, 52.00]	60.00 [48.00, 67.00]	<0.001
BMI	23.44 [20.96, 26.67]	23.15 [20.83, 26.49]	24.26 [21.98, 27.70]	0.009
anti-SARS-CoV-2 spike protein IgG (S/CO)	43.48 [6.88, 120.79]	43.06 [5.72, 95.73]	161.83 [22.45, 423.50]	<0.001
WBC (×10^9^/L)	6.22 [4.94, 7.73]	6.10 [4.90, 7.57]	6.74 [5.28, 9.12]	0.006
NE (×10^9^/L)	3.95 [2.93, 5.40]	3.80 [2.87, 5.11]	4.82 [3.31, 6.93]	<0.001
LY (×10^9^/L)	1.53 [0.99, 2.05]	1.58 [1.08, 2.08]	1.15 [0.78, 1.67]	<0.001
NLR	2.57 [1.68, 4.53]	2.42 [1.60, 3.94]	3.74 [2.28, 8.77]	<0.001
Monocyte (×10^9^/L)	0.54 [0.38, 0.79]	0.53 [0.35, 0.77]	0.78 [0.50, 0.97]	<0.001
Lac (mmol/L)	2.37 [1.55, 3.30]	2.34 [1.55, 3.26]	2.67 [1.55, 3.62]	0.159
CRP (mg/L)	4.65 [1.02, 16.05]	3.40 [0.80, 13.07]	16.35 [5.72, 47.90]	<0.001
LDH (U/L)	189.90 [161.70, 231.50]	184.60 [159.45, 215.78]	249.00 [204.67, 366.33]	<0.001
CK (U/L)	64.45 [42.42, 97.90]	66.50 [44.30, 100.08]	57.40 [32.32, 83.10]	0.004
CK-MB (U/L)	14.00 [10.72, 17.98]	13.80 [10.70, 17.50]	14.80 [10.80, 19.27]	0.074
HD (U/L)	155.00 [134.00, 192.75]	151.00 [132.25, 177.00]	204.50 [154.00, 257.75]	<0.001
SAA (mg/L)	18.50 [5.03, 134.50]	14.70 [4.90, 97.17]	69.10 [10.60, 379.03]	<0.001
IL-6 (pg/mL)	7.37 [3.11, 16.16]	7.18 [2.70, 14.62]	14.27 [5.85, 60.47]	<0.001
ESR (mm/h)	20.00 [9.00, 35.00]	19.00 [8.00, 33.00]	35.00 [20.00, 62.00]	<0.001
T-pct (%)	65.24 [55.77, 75.58]	64.69 [55.77, 74.61]	67.12 [52.27, 79.26]	0.212
T-abs (cells/μL)	1335.00 [707.00, 1998.00]	1305.50 [666.00, 1957.00]	1633.00 [779.00, 2007.00]	0.035
CD8-pct (%)	27.10 [19.40, 34.81]	27.04 [18.39, 34.69]	28.32 [22.85, 37.08]	0.026
CD8-abs (cells/μL)	611.00 [339.00, 939.00]	611.00 [345.00, 939.00]	553.50 [295.50, 960.00]	0.542
CD4-pct (%)	38.65 [28.11, 49.50]	38.81 [28.40, 48.74]	37.89 [25.98, 50.86]	0.563
CD4-abs (cells/μL)	811.00 [292.00, 1209.00]	811.00 [308.75, 1207.00]	817.50 [165.25, 1225.50]	0.734
CD4-CD8-ratio	1.60 [0.99, 2.29]	1.56 [0.92, 2.28]	1.87 [1.21, 2.65]	0.006
NK-pct (%)	14.85 [6.44, 27.00]	13.96 [5.27, 24.54]	27.00 [17.66, 27.00]	<0.001
NK-abs (cells/μL)	278.00 [109.00, 493.00]	267.00 [99.00, 488.00]	390.00 [133.00, 611.00]	0.005
B-pct (%)	11.00 [5.14, 20.00]	13.00 [5.14, 21.00]	6.31 [4.63, 14.00]	<0.001
B-abs (cells/μL)	257.00 [175.50, 394.00]	257.00 [152.50, 404.00]	256.00 [191.00, 380.50]	0.962

Abbreviations: BMI, body mass index; IgG, anti-SARS-CoV-2 spike protein immunoglobulin G reported as dimensionless S/CO; WBC, white blood cell count; NE, neutrophil count; LY, lymphocyte count; NLR, neutrophil-to-lymphocyte ratio; Lac, lactate; CRP, C-reactive protein; LDH, lactate dehydrogenase; CK, creatine kinase; CK-MB, creatine kinase isoenzyme MB; HD, α-hydroxybutyrate dehydrogenase; SAA, serum amyloid A; IL-6, interleukin-6; ESR, erythrocyte sedimentation rate; T-pct, T cell percentage; T-abs, T cell absolute count; CD8-pct, CD8^+^ T cell percentage; CD8-abs, CD8^+^ T cell absolute count; CD4-pct, CD4^+^ T cell percentage; CD4-abs, CD4^+^ T cell absolute count; NK-pct, NK cell percentage; NK-abs, NK cell absolute count; B-pct, B cell percentage; B-abs, B cell absolute count.

**Table 2:** Multivariable logistic regression analysis of candidate predictors.

Variable	Beta	SE	z	OR	Lower-CI	Upper-CI	*p*
(Intercept)	−7.624	1.749	−4.358	0.000	0.000	0.015	<0.001
BMI	0.008	0.047	0.165	1.008	0.919	1.105	0.869
Sex	0.171	0.423	0.405	1.187	0.518	2.718	0.686
Smoking	0.517	0.459	1.126	1.677	0.682	4.125	0.26
Hypertension	0.902	0.459	1.963	2.463	1.001	6.06	0.05
Age	0.026	0.012	2.168	1.027	1.003	1.051	0.03
IgG	0.006	0.001	5.429	1.006	1.004	1.009	<0.001
WBC	0.3	0.347	0.865	1.35	0.684	2.666	0.387
NE	−0.236	0.379	−0.622	0.79	0.375	1.661	0.534
LY	0.313	0.353	0.886	1.367	0.685	2.728	0.375
NLR	0.103	0.057	1.787	1.108	0.99	1.24	0.074
Monocyte	1.458	0.707	2.063	4.296	1.075	17.167	0.039
CRP	0.011	0.008	1.458	1.011	0.996	1.026	0.145
LDH	0.013	0.005	2.905	1.013	1.004	1.023	0.004
CK	−0.004	0.002	−1.659	0.996	0.992	1.001	0.097
HD	−0.008	0.005	−1.603	0.992	0.983	1.002	0.109
SAA	−0.001	0.002	−0.631	0.999	0.996	1.002	0.528
IL-6	0.009	0.004	2.039	1.009	1	1.018	0.041
ESR	0.014	0.008	1.847	1.014	0.999	1.029	0.065
T-abs	0.001	0	1.356	1.001	1	1.001	0.175
CD8-pct	−0.06	0.024	−2.51	0.942	0.899	0.987	0.012
CD4-CD8-ratio	−0.294	0.234	−1.255	0.745	0.471	1.18	0.209
NK-pct	0.103	0.021	4.964	1.109	1.065	1.155	<0.001
NK-abs	−0.004	0.001	−2.706	0.996	0.993	0.999	0.007
B-pct	−0.116	0.034	−3.388	0.891	0.833	0.952	0.001

Abbreviations: BMI, body mass index; CD4/CD8-ratio, CD4/CD8 ratio; CD8-pct, CD8^+^ T cell percentage; CI, confidence interval; CK, creatine kinase; CRP, C-reactive protein; ESR, erythrocyte sedimentation rate; HD, α-hydroxybutyrate dehydrogenase; IgG, anti-SARS-CoV-2 spike protein immunoglobulin G reported as dimensionless S/CO; IL-6, interleukin-6; LDH, lactate dehydrogenase; LY, lymphocyte count; NE, neutrophil count; NK-abs, NK cell absolute count; NK-pct, NK cell percentage; NLR, neutrophil-to-lymphocyte ratio; OR, odds ratio; SAA, serum amyloid A; SE, standard error; T-abs, T cell absolute count; WBC, white blood cell count.

**Table 3:** Discriminative performance of the CORIS nomogram versus individual biomarkers.

Dataset	Marker	Optimal-Cutoff	AUROC-95-CI	Accuracy	Sensitivity	Specificity	Precision	F1-Score	Kappa	Youdens-J	PPV	NPV
Training Set												
	CORIS	0.133	0.914 (0.884–0.94)	0.834 (0.801–0.863)	0.856 (0.768–0.914)	0.830 (0.793–0.862)	0.500	0.631	0.534	0.686	0.500	0.967
	LDH	206.000	0.772 (0.713–0.825)	0.707 (0.668–0.744)	0.756 (0.658–0.833)	0.698 (0.654–0.738)	0.332	0.461	0.300	0.453	0.332	0.935
	Age	53.500	0.77 (0.721–0.819)	0.762 (0.725–0.796)	0.711 (0.610–0.795)	0.773 (0.732–0.809)	0.383	0.498	0.360	0.484	0.383	0.931
	NK-pct	19.240	0.727 (0.665–0.789)	0.685 (0.645–0.723)	0.733 (0.634–0.814)	0.675 (0.631–0.717)	0.310	0.436	0.264	0.409	0.310	0.927
	IgG	202.520	0.696 (0.621–0.771)	0.873 (0.842–0.898)	0.444 (0.346–0.547)	0.958 (0.935–0.973)	0.678	0.537	0.467	0.403	0.678	0.897
	IL-6	20.175	0.63 (0.555–0.696)	0.785 (0.748–0.817)	0.411 (0.315–0.514)	0.859 (0.824–0.888)	0.366	0.387	0.257	0.270	0.366	0.880
Validation Set												
	CORIS	0.238	0.918 (0.868–0.96)	0.870 (0.82–0.907)	0.842 (0.696–0.926)	0.876 (0.822–0.915)	0.571	0.681	0.603	0.718	0.571	0.966
	Age	50.500	0.779 (0.696–0.848)	0.814 (0.759–0.859)	0.632 (0.473–0.766)	0.850 (0.793–0.893)	0.350	0.475	0.324	0.467	0.350	0.934
	LDH	239.200	0.771 (0.67–0.861)	0.732 (0.671–0.785)	0.737 (0.580–0.850)	0.731 (0.664–0.788)	0.453	0.527	0.415	0.481	0.453	0.921
	IL-6	17.355	0.718 (0.609–0.813)	0.758 (0.698–0.808)	0.605 (0.447–0.744)	0.788 (0.725–0.839)	0.385	0.444	0.314	0.361	0.385	0.899
	NK-pct	26.865	0.701 (0.598–0.8)	0.805 (0.749–0.851)	0.579 (0.422–0.721)	0.850 (0.793–0.893)	0.359	0.451	0.308	0.393	0.359	0.910
	IgG	154.083	0.674 (0.564–0.784)	0.784 (0.726–0.832)	0.526 (0.373–0.675)	0.834 (0.775–0.880)	0.431	0.494	0.377	0.429	0.431	0.911

Abbreviations: CORIS, COVID Routine-Immune Score nomogram; AUC, area under the receiver operating characteristic curve; CI, confidence interval; PPV, positive predictive value; NPV, negative predictive value; LDH, lactate dehydrogenase; IgG, anti-SARS-CoV-2 spike protein immunoglobulin G reported as dimensionless S/CO; IL-6, interleukin-6; NK-pct, NK cell percentage.

**Table 4:** Discriminative performance of the CORIS nomogram, ablated models, and previously published models in the internal validation set.

Model	CORIS	CORIS Without IL-6	CORIS Without NK-pct	Li 2021	Chen 2024
Variables	Age + LDH + IgG + IL-6 + NK-pct	Age + LDH + IgG + NK-pct	Age + LDH + IgG + IL-6	Sex + Age + T-abs + B-abs + CD4-abs	Age + NE + LDH + LY + CRP (ALB substituted)
Cutoff	0.238	0.260	0.144	0.151	0.181
AUC (95% CI)	0.918 (0.868–0.960)	0.907 (0.856–0.950)	0.885 (0.814–0.945)	0.811 (0.745–0.872)	0.839 (0.768–0.901)
Accuracy	0.870 (0.821–0.907)	0.866 (0.816–0.904)	0.792 (0.735–0.840)	0.732 (0.671–0.785)	0.814 (0.759–0.859)
Sensitivity	0.842 (0.696–0.926)	0.842 (0.696–0.926)	0.868 (0.727–0.942)	0.789 (0.637–0.889)	0.763 (0.608–0.870)
Specificity	0.876 (0.822–0.915)	0.870 (0.816–0.911)	0.777 (0.713–0.830)	0.720 (0.653–0.779)	0.824 (0.764–0.871)
Precision	0.571	0.561	0.434	0.357	0.46
F1-Score	0.681	0.674	0.579	0.492	0.574
Kappa	0.603	0.593	0.461	0.343	0.464
Youden-J	0.718	0.713	0.646	0.510	0.587
PPV	0.571	0.561	0.434	0.357	0.46
NPV	0.966	0.966	0.968	0.946	0.946
DeLong *p*	-	0.018	0.013	<0.001	0.003

Abbreviations: CORIS, COVID Routine-Immune Score nomogram; AUC, area under the receiver operating characteristic curve; CI, confidence interval; PPV, positive predictive value; NPV, negative predictive value; LDH, lactate dehydrogenase; IgG, anti-SARS-CoV-2 spike protein immunoglobulin G reported as dimensionless S/CO; IL-6, interleukin-6; NK-pct, NK cell percentage; T-abs, T cell absolute count; CD4-abs, CD4^+^ T cell absolute count; B-abs, B cell absolute count; NE, neutrophil count; LY, lymphocyte count; CRP, C-reactive protein; ALB, albumin.

## Data Availability

Since this dataset comes from retrospective data at Beijing Ditan Hospital of Capital Medical University, the dataset analyzed in this study is not publicly available, but it can be obtained from the corresponding author upon reasonable request.

## Supplementary Materials

The following supporting information can be downloaded at: https://www.mdpi.com/article/10.3390/diagnostics16193133/s1, Table S1: Missing ratio of variables in the study; Table: S2 Baseline characteristics of the training set; Table S3: Baseline characteristics of the validation set; Table S4: Comparison of baseline characteristics between the training and validation sets; Table S5: Variance Inflation factor test; Table S6: Nested cross-validation; Table S7: Multivariable logistic regression results of the five core predictors; Table S8: Overall performance and calibration of the CORIS model; Table S9: Sensitivity analyses adding BMI or vaccination status to the CORIS model; Figure S1: Correlation heatmap of candidate variables; Figure S2: Flowchart of the feature selection and model development pipeline; Figure S3: Performance evaluation of the CORIS nomogram in the training set. Clinical Case Demonstration.

## Abbreviations

AUC: Area Under the Receiver Operating Characteristic Curve; B-abs: B-cell absolute count; B-pct: B cell percentage; BMI: Body Mass Index; C: Classifier hyperparameter selected by inner-loop cross-validation; CD4-abs: CD4^+^ T cell absolute count; CD4-pct: CD4^+^ T cell percentage; CD8-abs: CD8^+^ T cell absolute count; CD8-pct: CD8^+^ T cell percentage; CD4/CD8-ratio: CD4/CD8 ratio; CI: Confidence Interval; CK: Creatine Kinase; CK-MB: Creatine Kinase Isoenzyme MB; CORIS: COVID Routine-Immune Score nomogram; CRP: C-reactive Protein; DCA: Decision Curve Analysis; ESR: Erythrocyte Sedimentation Rate; HD: α-Hydroxybutyrate Dehydrogenase; IgG: anti-SARS-CoV-2 spike protein immunoglobulin G reported as dimensionless S/CO; IL-6: Interleukin-6; Lac: Lactate; LASSO: Least Absolute Shrinkage and Selection Operator; LDH: Lactate Dehydrogenase; LY: Lymphocyte Count; NE: Neutrophil Count; NK-abs: NK cell absolute count; NK-pct: NK cell percentage; NLR: Neutrophil-to-Lymphocyte Ratio; NPV: Negative Predictive Value; OR: Odds Ratio; PPV: Positive Predictive Value; RFE: Recursive Feature Elimination; ROC: Receiver Operating Characteristic; SAA: Serum Amyloid A; SE: Standard Error; T-abs: T cell absolute count; T-pct: T cell percentage; VIF: Variance Inflation Factor; WBC: White Blood Cell Count.
