# Supplementary material for: Integrating Anti-SARS-CoV-2 Spike Protein IgG, IL-6, and NK Cell Percentage with Routine Laboratory Markers for COVID-19 Severity Assessment: Development and Validation of the CORIS Nomogram
Source: Diagnostics (Basel). 2026 Sep 26;16(19):3133. doi: 10.3390/diagnostics16193133 (PMC13664150; doi:10.3390/diagnostics16193133)
Supplement: Supplementary file 1 [file diagnostics-16-03133-s001.zip › diagnostics-4565015-supplementary.pdf]

Integrating Anti-SARS-CoV-2 Spike Protein IgG, IL-6, and NK Cell Percentage With Routine Laboratory Markers for COVID-19 Severity Assessment: Development and Validation of the CORIS Nomogram

Yihe Zhang [a,b,#], Xuanming Xu [a,#], Lian Tang [c], Jiayue Ma [a], Yajie Wang [a,*]

[a] National Key Laboratory of Intelligent Tracking and Forecasting for Infectious Diseases, Beijing Key Laboratory of Viral Infectious Diseases, Department of Clinical Laboratory, Beijing Ditan Hospital, Capital Medical University, Beijing 100015, China

[b] School of Medical Technology, Capital Medical University, Beijing 101300, China

[c] Department of Neurology, Beijing Tiantan Hospital, Capital Medical University, Beijing 100070, China

[#] These authors equally contributed to this work

[*] Corresponding Author: Yajie Wang

Directory

Table S1 Missing ratio of variables in the study.

Table S2 Baseline characteristics of the training set.

Table S3 Baseline characteristics of the validation set.

Table S4 Comparison of baseline characteristics between the training and validation sets.

Table S5 Variance Inflation factor test.

Table S6 Nested cross-validation.

Table S7 Multivariable logistic regression results of the five core predictors.

Table S8 Overall performance and calibration of the CORIS model.

Table S9 Sensitivity analyses adding BMI or vaccination status to the CORIS model.

Figure S1 Correlation heatmap of candidate variables.

Figure S2 Flowchart of the feature selection and model development pipeline.

Figure S3 Performance evaluation of the CORIS nomogram in the training set.

Clinical Case Demonstration

Table S1 Missing ratio of variables in the study.

Variable	Missing Ratio (%)
Outcome	0
Sex	0
Smoking	0
Alcohol	0
Diabetes	0
Hypertension	2.94
Hyperlipidemia	3.87
Age	0
BMI	0
anti-SARS-CoV-2 spike protein IgG	9.75
WBC	0
NE	0
LY	0
NLR	0
Monocyte	0
Lac	0
CRP	3.41
LDH	1.55
CK	0
CK-MB	0
HD	0
SAA	0
IL-6	0
ESR	0
T-pct	3.72
T-abs	0
CD8-pct	0
CD8-abs	0
CD4-pct	0
CD4-abs	0
CD4-CD8-ratio	0
NK-pct	0
NK-abs	0
B-pct	0
B-abs	5.11

Table S2 Baseline characteristics of the training set.

Variables	Total(n=543)	Non-severe(n=453)	Severe(n=90)	*P*
Vaccination status, n (%)				0.301
Yes	495 (91.16)	416 (91.83)	79 (87.78)	
No	48 (8.84)	37 (8.17)	11 (12.22)	
Sex				0.026
Male	289 (53.22)	231 (50.99)	58 (64.44)	
Female	254 (46.78)	222 (49.01)	32 (35.56)	
Smoking				0.046
Yes	117 (21.55)	90 (19.87)	27 (30.00)	
No	426 (78.45)	363 (80.13)	63 (70.00)	
Alcohol				0.911
Yes	142 (26.15)	118 (26.05)	24 (26.67)	
No	401 (73.85)	335 (73.95)	66 (73.33)	
Diabetes				0.717
Yes	290 (53.41)	244 (53.86)	46 (51.11)	
No	253 (46.59)	209 (46.14)	44 (48.89)	
Hypertension				0.001
Yes	332 (61.14)	262 (57.84)	70 (77.78)	
No	211 (38.86)	191 (42.16)	20 (22.22)	
Hyperlipidemia				0.551
Yes	271 (49.91)	223 (49.23)	48 (53.33)	
No	272 (50.09)	230 (50.77)	42 (46.67)	
Age	42.00 [31.00, 57.00]	39.00 [29.00, 52.00]	60.50 [48.00, 66.00]	<0.001
BMI	23.67 [21.03, 26.69]	23.38 [20.96, 26.56]	24.56 [22.11, 27.92]	0.03
anti-SARS-CoV-2 spike protein IgG (S/CO)	43.48 [7.06, 112.90]	43.06 [5.72, 91.93]	146.44 [26.03, 406.58]	<0.001
WBC ($\times10^9$/L)	6.27 [5.00, 7.86]	6.25 [5.00, 7.82]	6.56 [5.21, 8.41]	0.252
NE ($\times10^9$/L)	4.02 [2.96, 5.47]	3.97 [2.88, 5.33]	4.30 [3.25, 6.65]	0.014
LY ($\times10^9$/L)	1.56 [1.04, 2.05]	1.62 [1.14, 2.08]	1.15 [0.80, 1.67]	<0.001
NLR	2.56 [1.65, 4.38]	2.46 [1.60, 3.85]	3.58 [2.14, 7.92]	<0.001
Monocyte ($\times10^9$/L)	0.57 [0.38, 0.82]	0.54 [0.37, 0.77]	0.78 [0.46, 0.97]	<0.001
Lac (mmol/L)	2.38 [1.55, 3.31]	2.37 [1.55, 3.28]	2.71 [1.62, 3.62]	0.313
CRP (mg/L)	4.60 [1.00, 15.85]	3.30 [0.80, 13.10]	14.50 [4.95, 43.15]	<0.001
LDH (U/L)	189.90 [162.85, 230.05]	184.30 [159.60, 213.30]	247.75 [206.38, 324.80]	<0.001
CK (U/L)	63.70 [43.00, 94.80]	64.70 [44.40, 97.80]	57.40 [33.30, 81.97]	0.008
CK-MB (U/L)	14.00 [10.40, 17.85]	13.80 [10.20, 17.40]	14.65 [10.80, 19.05]	0.135
HD (U/L)	155.00 [135.50, 190.00]	152.00 [133.00, 177.00]	199.50 [152.00, 244.50]	<0.001
SAA (mg/L)	17.40 [5.30, 121.95]	15.30 [5.10, 89.70]	60.55 [9.50, 373.80]	<0.001
IL-6 (pg/mL)	7.33 [2.90, 16.16]	7.14 [2.66, 15.21]	13.39 [5.18, 51.49]	<0.001
ESR (mm/h)	20.00 [10.00, 35.00]	19.00 [8.00, 33.00]	34.00 [20.00, 62.00]	<0.001
T-pct (%)	65.24 [55.77, 75.46]	64.69 [55.77, 73.47]	68.79 [53.55, 79.01]	0.169
T-abs (cells/μL)	1335.00 [666.00, 1998.00]	1301.00 [666.00, 1957.00]	1605.00 [691.25, 1998.00]	0.238
CD8-pct (%)	27.12 [19.50, 34.97]	27.12 [18.61, 34.81]	27.56 [22.51, 36.65]	0.183
CD8-abs (cells/μL)	607.00 [345.00, 939.00]	611.00 [349.00, 939.00]	555.50 [292.50, 880.00]	0.349
CD4-pct (%)	37.79 [27.65, 47.96]	37.79 [28.11, 46.15]	38.97 [25.84, 50.86]	0.437
CD4-abs (cells/μL)	811.00 [292.00, 1201.00]	811.00 [329.00, 1201.00]	792.50 [163.75, 1209.00]	0.493
CD4-CD8-ratio	1.59 [0.92, 2.18]	1.53 [0.92, 2.18]	1.89 [1.15, 2.67]	0.007
NK-pct (%)	14.70 [6.79, 26.73]	13.93 [5.96, 23.32]	27.00 [18.25, 27.00]	<0.001
NK-abs (cells/μL)	267.00 [99.00, 488.00]	254.00 [99.00, 455.00]	365.00 [109.00, 512.00]	0.051
B-pct (%)	11.59 [5.14, 20.00]	13.26 [5.14, 21.00]	7.00 [4.72, 14.85]	<0.001
B-abs (cells/μL)	257.00 [153.00, 404.00]	257.00 [152.00, 413.00]	256.00 [185.25, 358.00]	0.409

Table S3 Baseline characteristics of the validation set.

Variables	Total(n=231)	Non-severe(n=193)	Severe(n=38)	*P*
Vaccination status, n (%)				0.247
Yes	214 (92.64)	181 (93.78)	33 (86.84)	
No	17 (7.36)	12 (6.22)	5 (13.16)	
Sex				0.001
Male	121 (52.38)	91 (47.15)	30 (78.95)	
Female	110 (47.62)	102 (52.85)	8 (21.05)	
Smoking				0.044
Yes	53 (22.94)	39 (20.21)	14 (36.84)	
No	178 (77.06)	154 (79.79)	24 (63.16)	
Alcohol				0.044
Yes	48 (20.78)	35 (18.13)	13 (34.21)	
No	183 (79.22)	158 (81.87)	25 (65.79)	
Diabetes				0.662
Yes	126 (54.55)	107 (55.44)	19 (50.00)	
No	105 (45.45)	86 (44.56)	19 (50.00)	
Hypertension				0.025
Yes	129 (55.84)	101 (52.33)	28 (73.68)	
No	102 (44.16)	92 (47.67)	10 (26.32)	
Hyperlipidemia				0.702
Yes	119 (51.52)	101 (52.33)	18 (47.37)	
No	112 (48.48)	92 (47.67)	20 (52.63)	
Age	40.00 [30.00, 58.00]	37.00 [28.00, 51.00]	59.50 [48.75, 68.00]	<0.001
BMI	22.86 [20.75, 26.41]	22.66 [20.55, 26.12]	23.84 [21.86, 27.16]	0.116
anti-SARS-CoV-2 spike protein IgG (S/CO)	48.78 [5.98, 135.30]	43.48 [5.80, 106.18]	195.72 [10.78, 470.06]	0.001
WBC ($\times 10^9$/L)	5.93 [4.90, 7.38]	5.83 [4.87, 7.14]	7.46 [5.47, 10.54]	0.001

NE (×10⁹/L)	3.70 [2.91, 5.14]	3.65 [2.84, 4.72]	5.31 [3.66, 7.08]	<0.001
LY (×10⁹/L)	1.44 [0.91, 2.01]	1.48 [0.97, 2.07]	1.15 [0.76, 1.67]	0.006
NLR	2.58 [1.75, 4.90]	2.30 [1.60, 4.14]	4.89 [2.67, 9.60]	<0.001
Monocyte (×10⁹/L)	0.53 [0.35, 0.76]	0.49 [0.33, 0.72]	0.78 [0.54, 0.96]	<0.001
Lac (mmol/L)	2.32 [1.43, 3.28]	2.27 [1.45, 3.18]	2.64 [1.43, 3.56]	0.32
CRP (mg/L)	5.60 [1.10, 16.50]	3.70 [0.80, 13.00]	20.45 [8.50, 70.60]	<0.001
LDH (U/L)	189.80 [160.70, 234.50]	185.40 [158.60, 222.30]	287.35 [202.75, 393.22]	<0.001
CK (U/L)	69.00 [40.85, 105.40]	69.50 [42.60, 103.60]	55.85 [32.02, 144.15]	0.291
CK-MB (U/L)	14.40 [11.05, 18.10]	13.80 [11.10, 17.60]	14.85 [11.00, 21.55]	0.349
HD (U/L)	154.00 [131.50, 197.50]	150.00 [129.00, 178.00]	237.50 [169.75, 269.00]	<0.001
SAA (mg/L)	19.90 [4.50, 152.85]	13.10 [4.20, 120.10]	91.65 [19.80, 391.95]	<0.001
IL-6 (pg/mL)	7.62 [3.46, 16.20]	7.24 [3.19, 13.82]	18.49 [7.20, 72.46]	<0.001
ESR (mm/h)	20.00 [6.00, 35.00]	16.00 [5.00, 33.00]	44.00 [20.25, 62.00]	<0.001
T-pct (%)	65.24 [55.77, 76.33]	66.16 [57.32, 75.58]	64.49 [49.62, 79.49]	0.851
T-abs (cells/μL)	1352.00 [756.00, 1998.00]	1315.00 [666.00, 1957.00]	1693.00 [933.25, 2017.00]	0.044
CD8-pct (%)	26.61 [18.39, 34.69]	25.49 [18.22, 34.46]	31.01 [24.30, 39.76]	0.043
CD8-abs (cells/μL)	612.00 [328.00, 939.00]	612.00 [328.00, 939.00]	553.00 [330.50, 1026.00]	0.692
CD4-pct (%)	40.44 [28.40, 49.92]	41.06 [28.56, 49.92]	37.12 [26.38, 51.26]	0.928
CD4-abs (cells/μL)	817.00 [292.00, 1242.00]	799.00 [292.00, 1209.00]	903.00 [274.00, 1302.75]	0.682
CD4-CD8-ratio	1.71 [1.01, 2.40]	1.71 [0.99, 2.40]	1.84 [1.24, 2.46]	0.379
NK-pct (%)	16.00 [5.27, 27.00]	14.00 [5.27, 25.00]	27.00 [14.00, 27.00]	<0.001
NK-abs (cells/μL)	293.00 [137.00, 493.00]	291.00 [130.00, 488.00]	417.00 [179.25, 611.00]	0.028
B-pct (%)	11.00 [5.00, 20.00]	12.36 [5.14, 21.00]	5.14 [4.72, 10.78]	0.001
B-abs (cells/μL)	256.00 [178.00, 394.00]	256.00 [177.00, 391.00]	287.00 [220.50, 404.00]	0.207

Table S4 Comparison of baseline characteristics between the training and validation sets.

Variables	Total(n=774)	Training set(n=543)	Validation set(n=231)	*P*
Vaccination status, n (%)				0.591
Yes	709 (91.60)	495 (91.16)	214 (92.64)	
No	65 (8.40)	48 (8.84)	17 (7.36)	
Sex				0.892
Male	410 (52.97)	289 (53.22)	121 (52.38)	
Female	364 (47.03)	254 (46.78)	110 (47.62)	
Smoking				0.738
Yes	170 (21.96)	117 (21.55)	53 (22.94)	
No	604 (78.04)	426 (78.45)	178 (77.06)	
Alcohol				0.134
Yes	190 (24.55)	142 (26.15)	48 (20.78)	
No	584 (75.45)	401 (73.85)	183 (79.22)	
Diabetes				0.832
Yes	416 (53.75)	290 (53.41)	126 (54.55)	
No	358 (46.25)	253 (46.59)	105 (45.45)	
Hypertension				0.196
Yes	461 (59.56)	332 (61.14)	129 (55.84)	
No	313 (40.44)	211 (38.86)	102 (44.16)	
Hyperlipidemia				0.741
Yes	390 (50.39)	271 (49.91)	119 (51.52)	
No	384 (49.61)	272 (50.09)	112 (48.48)	
Age	41.00 [31.00, 57.00]	42.00 [31.00, 57.00]	40.00 [30.00, 58.00]	0.495
BMI	23.44 [20.96, 26.67]	23.67 [21.03, 26.69]	22.86 [20.75, 26.41]	0.195
anti-SARS-CoV-2 spike protein IgG (S/CO)	43.48 [6.88, 120.79]	43.48 [7.06, 112.90]	48.78 [5.98, 135.30]	0.385
WBC (×10⁹/L)	6.22 [4.94, 7.73]	6.27 [5.00, 7.86]	5.93 [4.90, 7.38]	0.119
NE (×10⁹/L)	3.95 [2.93, 5.40]	4.02 [2.96, 5.47]	3.70 [2.91, 5.14]	0.257
LY (×10⁹/L)	1.53 [0.99, 2.05]	1.56 [1.04, 2.05]	1.44 [0.91, 2.01]	0.055
NLR	2.57 [1.68, 4.53]	2.56 [1.65, 4.38]	2.58 [1.75, 4.90]	0.662
Monocyte (×10⁹/L)	0.54 [0.38, 0.79]	0.57 [0.38, 0.82]	0.53 [0.35, 0.76]	0.105
Lac (mmol/L)	2.37 [1.55, 3.30]	2.38 [1.55, 3.31]	2.32 [1.43, 3.28]	0.152
CRP (mg/L)	4.65 [1.02, 16.05]	4.60 [1.00, 15.85]	5.60 [1.10, 16.50]	0.442
LDH (U/L)	189.90 [161.70, 231.50]	189.90 [162.85, 230.05]	189.80 [160.70, 234.50]	0.943
CK (U/L)	64.45 [42.42, 97.90]	63.70 [43.00, 94.80]	69.00 [40.85, 105.40]	0.268
CK-MB (U/L)	14.00 [10.72, 17.98]	14.00 [10.40, 17.85]	14.40 [11.05, 18.10]	0.384
HD (U/L)	155.00 [134.00, 192.75]	155.00 [135.50, 190.00]	154.00 [131.50, 197.50]	0.934
SAA (mg/L)	18.50 [5.03, 134.50]	17.40 [5.30, 121.95]	19.90 [4.50, 152.85]	0.966
IL-6 (pg/mL)	7.37 [3.11, 16.16]	7.33 [2.90, 16.16]	7.62 [3.46, 16.20]	0.353
ESR (mm/h)	20.00 [9.00, 35.00]	20.00 [10.00, 35.00]	20.00 [6.00, 35.00]	0.274
T-pct (%)	65.24 [55.77, 75.58]	65.24 [55.77, 75.46]	65.24 [55.77, 76.33]	0.934

T-abs (cells/μL)	1335.00 [707.00, 1998.00]	1335.00 [666.00, 1998.00]	1352.00 [756.00, 1998.00]	0.656
CD8-pct (%)	27.10 [19.40, 34.81]	27.12 [19.50, 34.97]	26.61 [18.39, 34.69]	0.606
CD8-abs (cells/μL)	611.00 [339.00, 939.00]	607.00 [345.00, 939.00]	612.00 [328.00, 939.00]	0.695
CD4-pct (%)	38.65 [28.11, 49.50]	37.79 [27.65, 47.96]	40.44 [28.40, 49.92]	0.103
CD4-abs (cells/μL)	811.00 [292.00, 1209.00]	811.00 [292.00, 1201.00]	817.00 [292.00, 1242.00]	0.662
CD4-CD8-ratio	1.60 [0.99, 2.29]	1.59 [0.92, 2.18]	1.71 [1.01, 2.40]	0.199
NK-pct (%)	14.85 [6.44, 27.00]	14.70 [6.79, 26.73]	16.00 [5.27, 27.00]	0.738
NK-abs (cells/μL)	278.00 [109.00, 493.00]	267.00 [99.00, 488.00]	293.00 [137.00, 493.00]	0.318
B-pct (%)	11.00 [5.14, 20.00]	11.59 [5.14, 20.00]	11.00 [5.00, 20.00]	0.313
B-abs (cells/μL)	257.00 [175.50, 394.00]	257.00 [153.00, 404.00]	256.00 [178.00, 394.00]	0.809

Table S5 Variance Inflation factor test.

**Variable**	**VIF**
Age	1.05
LDH	1.05
IgG	1.03
IL-6	1.05
NK-pct	1.01

Abbreviations: VIF, variance inflation factor; LDH, lactate dehydrogenase; IgG, anti-SARS-CoV-2 spike protein immunoglobulin G reported as dimensionless S/CO; IL-6, interleukin-6; NK-pct, NK cell percentage.

Table S6 Nested cross-validation.

**Fold**	**AUC**	**Best-C**	**Features**
1	0.913	0.01	('Age', 'IgG', 'NLR', 'CRP', 'LDH', 'IL-6', 'ESR', 'NK-pct')
2	0.913	0.01	('Age', 'IgG', 'NLR', 'CRP', 'LDH', 'IL-6', 'ESR', 'NK-pct')
3	0.914	10	('Age', 'IgG', 'CRP', 'LDH', 'CK', 'IL-6', 'ESR', 'NK-pct')
4	0.914	0.1	('Age', 'IgG', 'CRP', 'LDH', 'CK', 'IL-6', 'ESR', 'NK-pct')
5	0.897	10	('Age', 'IgG', 'NLR', 'CRP', 'LDH', 'CK', 'ESR', 'NK-pct')
6	0.915	0.01	('Age', 'IgG', 'NLR', 'CRP', 'LDH', 'CK', 'IL-6', 'NK-pct')
7	0.915	0.1	('Age', 'IgG', 'NLR', 'CRP', 'LDH', 'CK', 'IL-6', 'NK-pct')
8	0.915	0.01	('Age', 'IgG', 'NLR', 'CRP', 'LDH', 'CK', 'IL-6', 'NK-pct')
9	0.915	0.01	('Age', 'IgG', 'NLR', 'CRP', 'LDH', 'CK', 'IL-6', 'NK-pct')
10	0.915	0.01	('Age', 'IgG', 'NLR', 'CRP', 'LDH', 'CK', 'IL-6', 'NK-pct')

Abbreviations: RFE, recursive feature elimination; AUC, area under the receiver operating characteristic curve; C, classifier hyperparameter selected by inner-loop cross-validation; IgG, anti-SARS-CoV-2 spike protein immunoglobulin G reported as dimensionless S/CO; NLR, neutrophil-to-lymphocyte ratio; CRP, C-reactive protein; LDH, lactate dehydrogenase; IL-6, interleukin-6; ESR, erythrocyte sedimentation rate; NK-pct, NK cell percentage; CK, creatine kinase.

Table S7 Multivariable logistic regression results of the five core predictors.

Variable	Beta	SE	Wald	OR	Lower-CI	Upper-CI	*P*
(Intercept)	-8.317	0.77	-10.795	0	0	0.001	<0.001
Age	0.047	0.01	4.715	1.048	1.028	1.069	<0.001
IgG	0.006	0.001	5.708	1.006	1.004	1.007	<0.001
IL-6	0.008	0.003	2.354	1.008	1.001	1.015	0.019
NK-pct	0.067	0.014	4.858	1.069	1.041	1.099	<0.001
LDH	0.009	0.002	4.728	1.009	1.006	1.013	<0.001

Abbreviations: SE, standard error; OR, odds ratio; CI, confidence interval; IgG, anti-SARS-CoV-2 spike protein immunoglobulin G reported as dimensionless S/CO; IL-6, interleukin-6; NK-pct, natural killer cell percentage; LDH, lactate dehydrogenase.

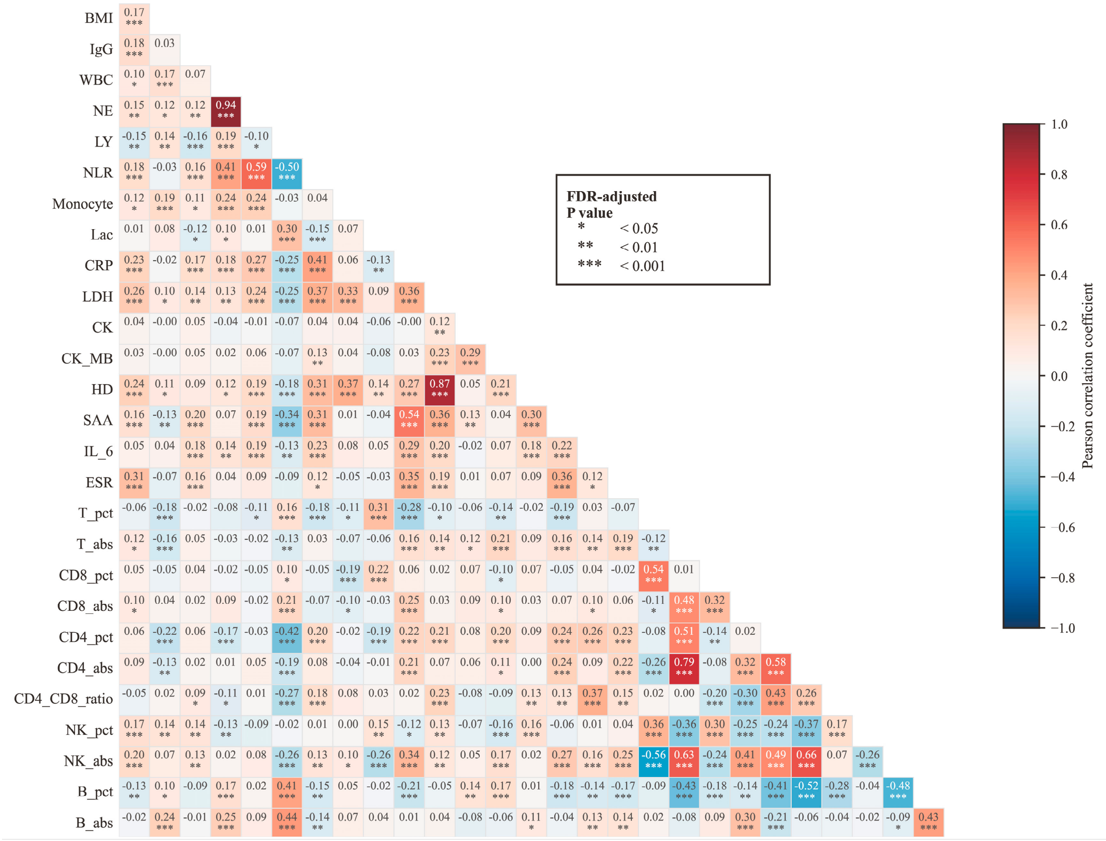

Table S8 Overall performance and calibration of the CORIS model.

Metric	Training set	Internal validation set
Participants, n	543	231
Severe/critical cases, n	90	38
Severe/critical proportion	0.166	0.165
AUC (95% CI)	0.914 (0.884–0.940)	0.918 (0.868–0.960)
Brier score	0.079	0.074
Calibration intercept (95% CI)	0.000 (-0.306–0.306)	-0.406 (-0.887–0.076)
Calibration slope (95% CI)	1.000 (0.812–1.188)	0.997 (0.714–1.279)
Evaluation role	Model development	Internal evaluation

Note: Calibration intercept and slope were estimated by logistic recalibration of the model linear predictor. Ideal values are 0 for the intercept and 1 for the slope. This was single-center model development with internal evaluation; larger multicenter external validation is required.

Table S9 Sensitivity analyses adding BMI or vaccination status to the CORIS model.

Model	Adjusted OR of added predictor (95% CI)	P	AUC (95% CI)	ΔAUC vs CORIS	DeLong P vs CORIS	Brier score
CORIS	—	—	0.918 (0.868–0.960)	—	—	0.074
CORIS plus BMI	0.993 (0.923–1.069)	0.855	0.919 (0.868–0.961)	+0.001	0.161	0.074
CORIS plus vaccination	0.687 (0.255–1.847)	0.457	0.920 (0.868–0.962)	+0.002	0.291	0.073

Note: Each model was fitted in the training set (n = 543) and evaluated in the internal validation set (n = 231; 38 severe/critical cases). The adjusted OR refers to the added predictor. AUC differences were compared with CORIS using DeLong's test.

Figure S1 Correlation heatmap of candidate variables.

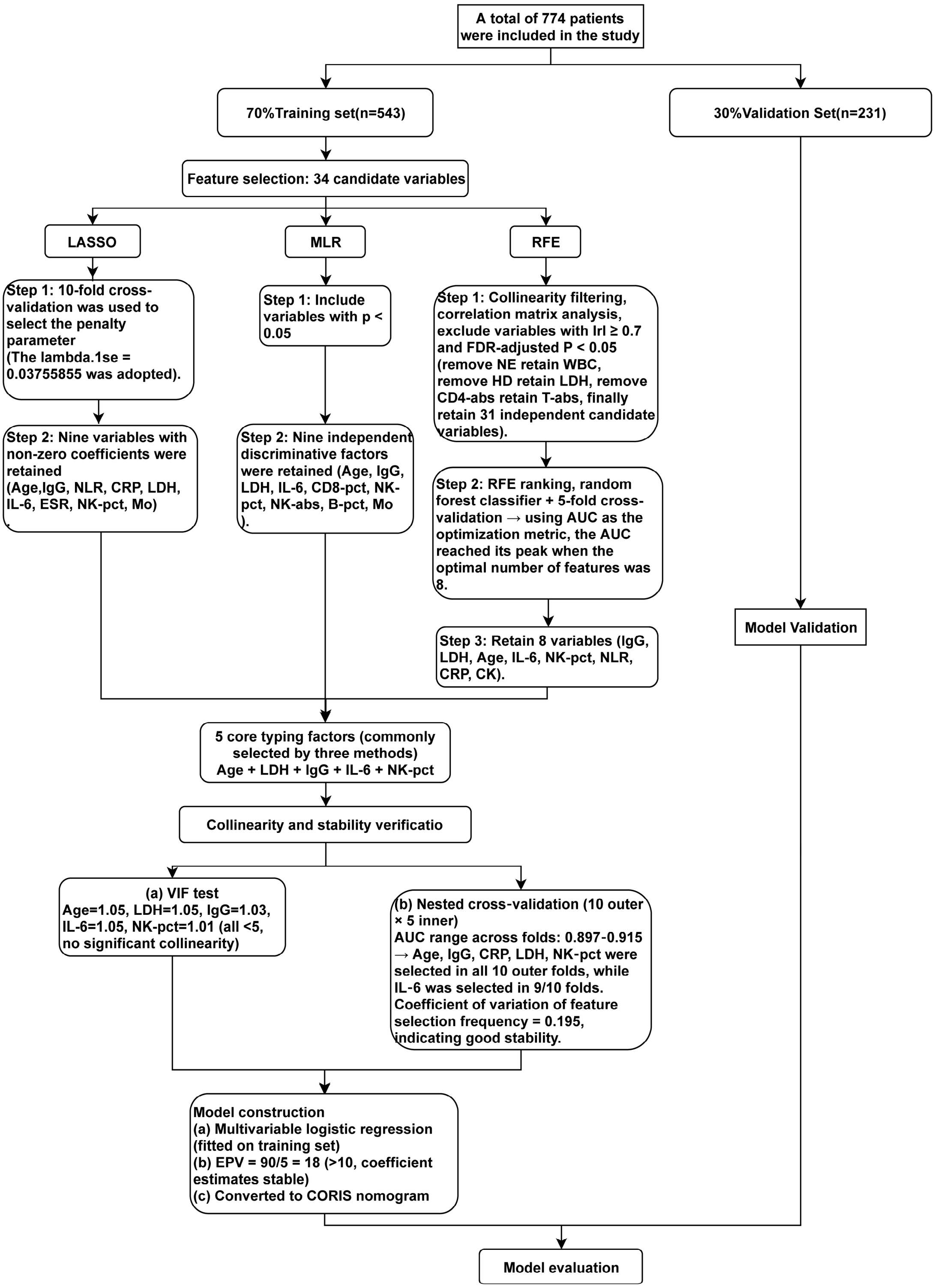

Figure S2 Flowchart of the feature selection and model development pipeline.

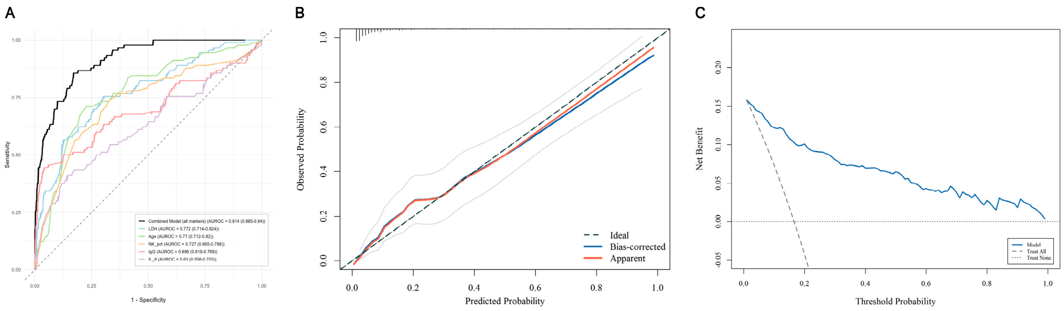

Figure S3 Performance evaluation of the CORIS nomogram in the training set. **(A)** ROC curve (AUC = 0.914, 95% CI by bootstrap internal resampling, 1000 iterations). **(B)** Calibration curve after bootstrap correction (H-L test: $\chi^2 = 9.2428$, df = 8, $P = 0.3222$). **(C)** Decision curve analysis.

Clinical Case Demonstration

Illustrative Use of the CORIS Nomogram for Admission Severity Assessment

The following cases illustrate calculation and interpretation of the probability of severe/critical status at admission only. They do not demonstrate prediction of subsequent deterioration, prognosis, or treatment response.

Case 1 (Non-Severe)

I. Case Background

A 27-year-old female presented with a 2-day history of fever, sore throat, and malaise. Nasopharyngeal swab tested positive for SARS-CoV-2 by nucleic acid amplification. She had no prior medical history of hypertension, diabetes, or other underlying conditions and had completed the full course of COVID-19 vaccination. The clinical team used the CORIS nomogram to estimate the probability of severe/critical status at admission as an adjunct to the formal clinical severity assessment and triage decision.

Core laboratory parameters at presentation:

- Age: 27 years
- Lactate dehydrogenase (LDH): 75 U/L
- anti-SARS-CoV-2 spike protein IgG: 85 S/CO
- Interleukin-6 (IL-6): 25 pg/mL
- NK cell percentage (NK-pct): 9%

II. Itemized Scoring of Individual Parameters

- Age: 27 years → approximately 10 points
- LDH: 75 U/L → approximately 5 points
- anti-SARS-CoV-2 spike protein IgG: 85 S/CO → approximately 4 points

- IL-6: 25 pg/mL → approximately 3 points

- NK-pct: 9% → approximately 6 points

III. Calculation of Composite Total Score

Total Points = 10 + 5 + 4 + 3 + 6 = 28 points

IV. Corresponding Outcome Risk Probability

A total score of 28 points corresponds to an estimated probability of severe/critical status at admission of <5%, indicating a low admission-severity probability.

V. Interpretation at Admission

1. Admission Severity Interpretation: The CORIS nomogram estimates a probability of severe/critical status at admission of <5% for this patient. When considered together with guideline-based clinical assessment, this result supports classification as non-severe at admission; it should not be interpreted as predicting subsequent disease progression.
2. Clinical Use: The result may support admission triage, but ward placement and resource allocation should be determined by contemporaneous clinical severity criteria and clinical judgment.
3. Subsequent Monitoring: Monitoring after admission should follow routine clinical practice. CORIS was not developed for serial recalculation or for prediction of later deterioration.
4. Precautions: The CORIS nomogram is an adjunct to admission severity assessment and cannot substitute for comprehensive clinical judgment or guideline-based severity criteria.

Case 2 (Severe/Critical)

I. Case Background

A 74-year-old male was admitted with a 5-day history of fever, cough, chest tightness, and shortness of breath. Nasopharyngeal swab tested positive for SARS-CoV-2 by nucleic acid amplification. His past medical history included essential hypertension and type 2 diabetes mellitus (12-year history) and chronic obstructive pulmonary disease (8-year history). On admission, his oxygen saturation on room air was 90% by pulse oximetry. The clinical team used the CORIS nomogram to estimate the probability of severe/critical status at admission as an adjunct to formal clinical severity assessment and triage.

Core laboratory parameters on admission:

- Age: 74 years

- Lactate dehydrogenase (LDH): 475 U/L

- anti-SARS-CoV-2 spike protein IgG: 300 S/CO

- Interleukin-6 (IL-6): 325 pg/mL

- NK cell percentage (NK-pct): 35%

II. Itemized Scoring of Individual Parameters

- Age: 74 years → approximately 31 points
- LDH: 475 U/L → approximately 30 points
- anti-SARS-CoV-2 spike protein IgG: 300 S/CO → approximately 16 points
- IL-6: 325 pg/mL → approximately 24 points
- NK-pct: 35% → approximately 20 points

III. Calculation of Composite Total Score

Total Points = 31 + 30 + 16 + 24 + 20 = 121 point

IV. Corresponding Outcome Risk Probability

A total score of 121 points corresponds to an estimated probability of severe/critical status at admission of >99%, indicating a high admission-severity probability.

V. Interpretation at Admission

1. Admission Severity Interpretation: The CORIS nomogram estimates a probability of severe/critical status at admission exceeding 99% for this patient. This result supports careful evaluation against formal admission severity criteria but does not predict the patient's subsequent clinical course.
2. Clinical Use: The level of care should be determined by guideline-based admission severity criteria and clinical judgment, not by the nomogram result alone.
3. Treatment Decisions: Treatment should follow current clinical guidelines and individualized assessment. CORIS was not developed to select antiviral, anti-inflammatory, or immunomodulatory treatment or to predict treatment response.
4. Subsequent Monitoring: Monitoring after admission should be based on the patient's clinical status. CORIS should not be serially recalculated to infer future deterioration without prospective validation.
5. Precautions: The CORIS nomogram supports severity discrimination at admission and cannot replace guideline-based clinical assessment. Its output should not be interpreted as a prognosis or as a recommendation for a specific treatment pathway.

Summary

The CORIS nomogram is an adjunctive tool for severity discrimination at hospital admission. It estimates the probability that a patient meets the severe/critical category at admission using a limited set of laboratory results. The output may support formal clinical assessment and triage, but it does not predict post-admission deterioration, prognosis, or treatment response and cannot replace guideline-based clinical judgment.
